# Chemoresistant fibroblasts dictate neoadjuvant chemotherapeutic response of head and neck cancer via TGFα-EGFR paracrine signaling

**DOI:** 10.1038/s41698-023-00460-2

**Published:** 2023-10-11

**Authors:** Liangping Su, Sangqing Wu, Cheng Huang, Xianhua Zhuo, Jiali Chen, Xue Jiang, Xiangzhan Kong, Cui Lv, Qiuping Xu, Ping Han, Xiaoming Huang, Ping-Pui Wong

**Affiliations:** 1 Guangdong Provincial Key Laboratory of Malignant Tumor Epigenetics and Gene Regulation, Guangdong-Hong Kong Joint Laboratory for RNA medicine, Sun Yat-sen Memorial Hospital, State Key Laboratory of Oncology in South China, Sun Yat-sen University, Guangzhou, 510120 China; 2 Medical Research Center, Sun Yat-sen Memorial Hospital, Sun Yat-sen University, Guangzhou, 510120 China; 3 Department of Otolaryngology, Head and Neck Surgery, Sun Yat-sen Memorial Hospital, Sun Yat-sen University, Guangzhou, 510120 China

**Keywords:** Cell biology, Cancer

## Abstract

Conventional chemotherapy targets malignant cells without evaluating counter protection from the tumor microenvironment that often causes treatment failure. Herein, we establish chemoresistant fibroblasts (rCAFs) as regulators of neoadjuvant chemotherapeutic (NACT) response in head and neck squamous cell carcinoma (HNSCC). Clinically, high expression of CAF-related gene signature correlates with worse prognosis and chemotherapeutic response in multiple cancers, while the population of CAFs in the residual tumors of chemoresistant HNSCC patients remains unchanged after NACT treatment, compared to chemosensitive patients. Using a murine cancer model or patient-derived organoid, and primary CAFs isolated from chemo-sensitive (sCAFs) or -resistant patients, we show that rCAFs, but not sCAFs, are resistant to chemotherapy-induced apoptosis while reducing HNSCC cell chemosensitivity via paracrine signals. Combined multi-omics and biochemical analyses indicate an elevated PI3K/AKT/p65 driven cell survival and cytokine production in rCAFs, while rCAF-secreted TGFα promotes cancer cell chemoresistance by activating EGFR/Src/STAT3 survival signaling axis. Treatment with anti-EGFR cetuximab restores the chemosensitivity of tumors derived from co-injection of cancer cells and rCAFs in vivo, while the serum level of TGFα determines NACT response in HNSCC patients. Overall, our findings uncover a novel insight whereby the crosstalk between tumor cell and rCAF determines chemotherapeutic response and prognosis in cancer patients.

## Introduction

Head and neck squamous cell carcinomas (HNSCC), originating from the oropharynx, oral cavity, larynx and hypopharynx, accounts for almost 90% of the hand and neck cancer cases, making it the sixth common malignancy by incidence in the world^1,2^. To tackle this issue, chemotherapy, such as fluorouracil (5’FU) or cisplatin (DDP) based treatment, is routinely given to HNSCC patients before or after surgery. The primary objective of neoadjuvant chemotherapy (NACT) treatment is resectability and organ preservation in advanced HNSCC patients^3–5^. However, resistance to standard chemotherapies has undermined the enormous efforts to improve patient prognosis in HNSCC^3,6,7^. Therefore, understanding the molecular basis of chemoresistance and identifying predictors of chemotherapeutic response are urgently needed to enhance the clinical outcomes in HNSCC.

The tumor microenvironment is a highly complex ecosystem consisting of different types of stromal cells including fibroblasts, blood vessels and immune cells as well as tumor cells themselves, while they interact and form a communication network to flourish tumor cell growth^8–10^. One crucial player in cancer progression and chemoresistance is cancer-associated fibroblasts (CAFs)^11^. Studies have shown that a functional subset of CAF, CD10^+^GPR77^+^ CAF, promotes breast cancer formation and chemoresistance by enriching cancer stemness^12^. Additionally, numerous studies have demonstrated a significant association between the proportion of α-SMA-expressing CAFs in tumors and chemoresistance in various cancers^13,14^. Despite this knowledge, researchers have failed to explain how CAFs survive chemotherapy treatment in resistant patients^15^, with one possibility being that chemotherapy may also affect the survival of CAFs via a poorly studied mechanism^16,17^. The direct crosstalk between tumor cells and fibroblasts plays a crucial role in cancer growth and progression^8,11,14^, but its role in modulating chemoresistance in HNSCC is not yet fully understood. Clinically approved drugs to disrupt this tumor-stromal crosstalk are currently unavailable, highlighting the importance of further research in this field.

Previous studies showed that the elevated epidermal growth factor receptor (EGFR) signaling axis contributes to the development and progression of HNSCC^18^, while 50–90% of HNSCC overexpresses EGFR^19^. Cetuximab is a monoclonal antibody that binds the extracellular domain of EGFR with higher affinity than its main ligands EGF and tumor growth factor-alpha (TGFα)^20^, which has been approved to be used in combination with chemotherapy or radiotherapy to treat HNSCC patients, mostly for those at advanced stage^12,21,22^. It is worth noting that in various biological systems, TGFα has been shown to be a more powerful agonist for EGFR than EGF^23,24^, indicating the potential importance of TGFα in regulating cancer progression. In first-line treatment, co-administration of cetuximab and cisplatin has shown to improve HNSCC patient response rate when compared with cisplatin alone^12,22^. However, the underlying mechanism behind this synergetic effect and the source of TGFα in the tumor microenvironment hasn’t been fully exploited. Consequently, there are no effective serum or tumor markers available to predict therapeutic responses in HNSCC patients undergoing combined cetuximab and cisplatin treatment. This highlights the unmet medical need to identify a subpopulation of HNSCC patients who can truly benefit from this specific treatment approach.

Here, we conducted a comprehensive study to determine the functional role of the interaction between tumor cells and CAFs in regulating chemoresistance in HNSCC. Our clinical investigations revealed a strong correlation between high CAF population, poor prognosis, and worse chemotherapeutic response in HNSCC patients. We found that the number of CAFs was higher in the residual tumors of chemoresistant patients after receiving NACT treatment compared with those of chemosensitive patients. To understand the underlying mechanisms, we developed a CAF isolation technique from pre-treatment HNSCC tumor biopsies and isolated CAFs from chemo-sensitive (sCAF) and -resistant (rCAF) patient-derived pre-treatment tumor biopsies, respectively. We discovered that rCAFs had a higher IC_50_ value for chemotherapeutic drugs compared with sCAFs. Transcriptomics and proteomics analysis showed a significant up-regulation of the PI3K/AKT/p65 signaling pathway in rCAFs compared with sCAFs, which promoted cell survival and cytokine production. The elevated PI3K/AKT/p65 signaling pathway also up-regulated TGFα secretion in rCAFs to reduce cancer cell chemosensitivity via activation of the EGFR/Src/STAT3-driven survival pathway and repression of the p53/caspase-3-driven apoptosis. We further demonstrated that rCAFs promoted cisplatin (DDP) resistance in HNSCC cancer cells, while administration of cetuximab restored cancer cell sensitivity to cisplatin in vitro and in vivo. Clinically, higher CAF-TGFα expression correlated with poor prognosis in HNSCC patients, while the serum level of TGF-α directly determined their therapeutic response to NACT. Our work highlighted the crucial role of the tumor cell-rCAF crosstalk in contributing to cancer chemoresistance through the TGFα-EGFR paracrine signaling pathway.

## Results

### Cancer-associated fibroblast population as a prognostic indicator of chemotherapeutic response in head and neck cancer patients

**Fig. 1:**
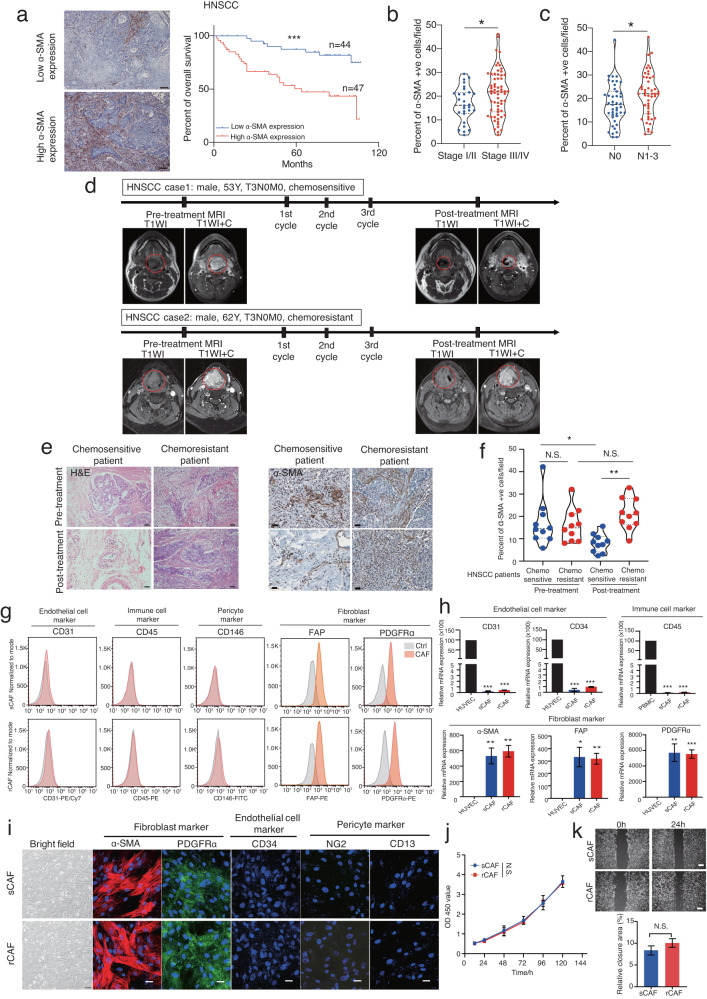
Cancer associated fibroblast population determines neoadjuvant chemotherapy efficacy in HNSCC. **a** Immunohistochemistry analysis of α-SMA expression in tumor sections derived from HNSCC patients. Kaplan–Meier survival curves of HNSCC patients with high or low α-SMA expression (*n* = 91 patients, our cohort). Violin plots depicting the relationship between cancer progression (**b**) or tumor size (**c**) and α-SMA expression level (*n* = 91 patients, our cohort). **d** Magnetic resonance imaging (MRI) of a chemo-sensitive or chemo-resistant patient before and after receiving neoadjuvant chemotherapy (NACT) treatment (3 cycles of treatment). Red line indicates the position of tumor. TNM staging: T stands for tumor, N for lymph node metastasis and M for metastasis. Y stands for year. T1Wl refers to T1 weighted images and T1Wl + C stands for contrast enhanced T1Wl. **e** Representative images of H&E and α-SMA immunohistochemical stained tumors sections from each group before and after the NACT treatment are given. **f** Violin plot showing the level of α-SMA expression in chemo-sensitive or -resistant HNSCC patients before and after receiving the NACT treatment (*n* = 20 patients, our cohort). **g** Flow cytometry (FACS) analysis of the expression levels of endothelial cell (CD31), immune cell (CD45), pericyte marker (CD146) and fibroblast markers (FAP, PDGFRα) in CAFs isolated from three different chemo-sensitive (sCAF) or -resistant (rCAF) patient derived pre-treatment tumor biopsies. **h** Analysis of the mRNA expression levels of endothelial cell, immune cell, pericyte and fibroblast markers in sCAFs and rCAFs respectively. **i** Immunofluorescence staining of the indicated endothelial cell, immune cell, pericyte and fibroblast markers in sCAFs and rCAFs. Representative bright field images of sCAF and rCAF are given. **j** CCK8 proliferation assay of the sCAFs and rCAFs over 120 h. **k** Representative images of scratch assays from each group. Bar charts show the percentage of wound closure in each group. FACS plots or bar charts are representative of three individual patient data. Data are presented as means ± S.E.M. *P < 0.05, **P < 0.01, ***P < 0.001, N.S. non-significant. Scale bars in (**a**) represent 100 μm, (**e**) 200 μm, (**i**, **k**) 50 μm. **a** Log-rank (Mantel-Cox) test. **b**, **c**, **k** Student’s *t* test. **f**, **h** One-way ANOVA. **j** Two-way ANOVA.

To determine whether CAF population is associated with patient prognosis, we performed an immunohistochemistry (IHC) examination of CAF-related marker expression, including α-SMA (alpha-smooth muscle actin) and PDGFRα (platelet derived growth factor receptor alpha), in our HNSCC patient cohort. Our data showed that higher expression of α-SMA or PDGFRα was correlated with worse overall survival, elevated cancer progression and enhanced lymph node metastasis (Fig. 1a–c, Supplementary Fig. 1a–c). We next sought to examine the contribution of CAFs to chemoresistance. The clinical samples derived from HNSCC patients before and after receiving NACT were harvested. Their response to NACT, including sensitive patients (CR: complete response, PR: partial response) or resistant patients (SD: stable disease and PD: progressive disease)), was monitored and evaluated effectively by magnetic resonance imaging (MRI) before and after the NACT treatment (Fig. 1d). By performing IHC staining of α-SMA and PDGFRα as well as H&E staining with these tissues, our results showed no significant difference in CAF population between sensitive and resistant patients before receiving NACT (Fig. 1e, f and Supplementary Fig. 1d, e). However, the number of CAFs was significantly reduced in the residual tumors of chemosensitive patients after treated with chemotherapy, as compared with the ones derived from chemoresistant patients (Fig. 1e, f and Supplementary Fig. 1d, e). Unlike chemosensitive patient derived tumors, there was no significant difference in CAF population for chemoresistant patient derived tumors before and after the NACT treatment (Fig. 1e, f and Supplementary Fig. 1d, e). To maximize the clinical importance of our findings, we utilized the online Kaplan-Meier (KM) plotter database tool that contained the clinical data of chemotherapy treated cancer patients, mainly from gastric and ovarian cancers (since they were frequently given chemotherapy treatment), to further examine the correlation between CAF gene signature expression and survival data. Our data also showed that higher α-SMA expression correlated with poor overall survival in 5’FU treated gastric cancer and platin alone or platin plus taxol treated ovarian cancer patients respectively (Supplementary Fig. 1f–h). Collectively, these results indicated that the population of CAFs determined chemotherapeutic responses in cancer patients.

To investigate the role of cancer associated fibroblasts in chemoresistance, we developed an effective method to isolate CAFs from neoadjuvant chemo-sensitive (sCAFs) and -resistant (rCAFs) HNSCC patient-derived pre-treatment tumor biopsies respectively (Supplementary Fig. 1i). The fibroblast identity of these isolated sCAFs and rCAFs was analyzed using flow cytometry (FCM) and RT-PCR as well as immunostaining experiments, which revealed that they both expressed PDGFRA (platelet derived growth factor alpha), the pericyte/fibroblast marker ACTA2 (α smooth muscle actin) and the fibroblast marker FAP (fibroblast associated protein), but not the endothelial cell marker CD34 and CD31, the pericyte makers CD146 (cluster of differentiation 146), the peripheral blood mononuclear cell (PBMC) marker CD45 and the epithelial cell marker EPCAM (epithelial cell adhesion molecule) as compared with human umbilical vein endothelial cells (HUVEC), PBMC, pericytes and HNSCC cells respectively (Fig. 1g–i, Supplementary Fig. 1j). We also assessed their cell morphology, cell proliferation and cell migration ability. Phase contrast microscope analysis indicated that both sCAFs and rCAFs possessed the morphological characteristics of fibroblasts, which were observed as flat and spindle shaped cells (Fig. 1i). Further functional studies revealed no significant differences in cell proliferation and migration between sCAFs and rCAFs (Fig. 1j, k). Together, our work developed a new protocol to isolate CAFs from NACT-resistant and-sensitive patient-derived pre-treatment tumor biopsies respectively, which can be then used for further functional and mechanistic studies.

### Cancer associated fibroblasts are functionally different in chemo-resistant and-sensitive HNSCC tumors

Having confirmed the fibroblast identity of our isolated sCAFs and rCAFs in the previous experiments, we investigated whether there were functional differences between the two groups in terms of chemotherapy resistance. Specifically, we sought to determine whether rCAFs were more resistant to chemotherapy, which could explain the observed increase in CAF population in residual tumors from chemoresistant patients after NACT treatment. Intriguingly, our IC_50_ experiments revealed that rCAFs exhibited higher resistance to cisplatin (DDP) or 5’FU as compared to sCAFs, (Supplementary Fig. 2a, b). Transwell migration and invasion assays showed that rCAFs exhibited stronger migration and invasion abilities in the presence of DDP or 5’FU when compared to sCAFs. However, there was no significant differences in migration and invasion ability between sCAFs and rCAFs when cultured in the medium without chemotherapeutic drugs (Supplementary Fig. 2c–f). These results provided the first evidence that rCAFs are more resistant to chemotherapy than sCAFs, which could account for the unaltered population of CAFs in chemoresistant patient before and after NACT treatment.

**Fig. 2:**
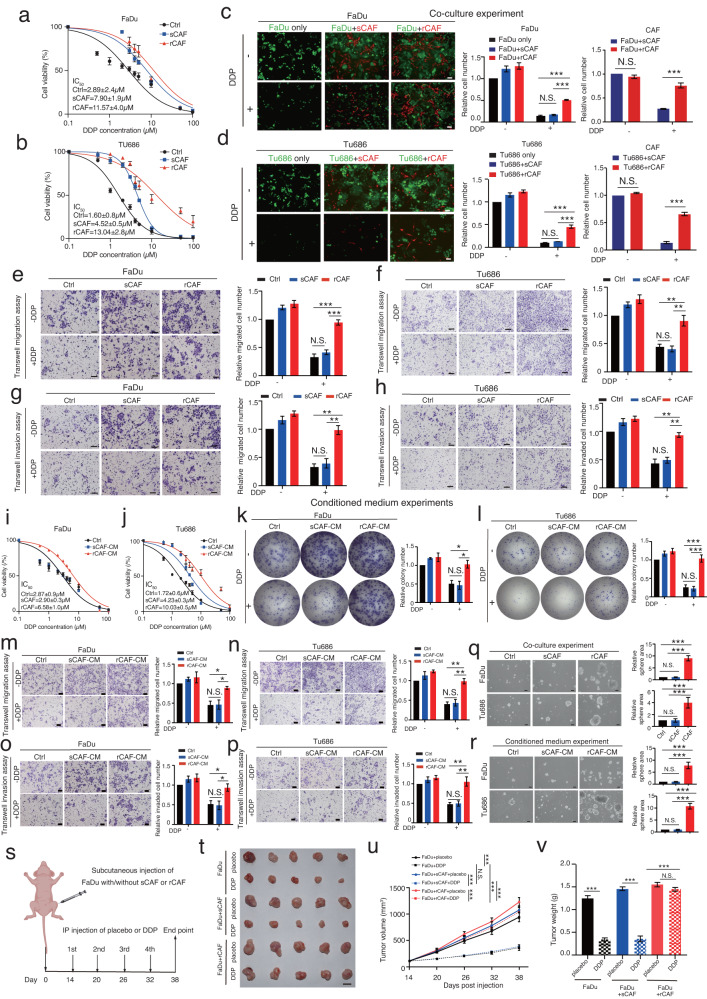
The paracrine effect of rCAFs, but not sCAFs, promotes cancer cell chemoresistance. **a**, **b** DDP IC_50_ experiments of FaDu or Tu686 cells after co-culture with or without sCAFs or rCAFs (*n* = 3 independent experiments). **c**, **d** Co-culturing GFP overexpressing FaDu or Tu686 cells with RFP expressing sCAFs or rCAFs for 48 h. Bar charts represent the relative number of cancer cells or CAFs in each group (*n* = 3 independent samples). Each result is shown after being normalized to the control in each experiment. **e**, **f** Migration assays of FaDu or Tu686 cells co-culture with sCAFs or rCAFs. Bar charts present the relative number of migrated cells in each group. **g**, **h** Invasion assays of FaDu or Tu686 cells co-cultured with sCAFs or rCAFs (*n* = 3 independent samples). **i**, **j** DDP IC_50_ experiments of FaDu or Tu686 cells in the presence or absence of conditioned medium (CM) from sCAFs or rCAFs. Untreated cancer cells were used as a control (ctrl). **k**, **l** Colony formation assays of FaDu or Tu686 cells after treated with/without CM from sCAFs or rCAFs. **m**, **n** Bar charts show the relative migrated cell number in each group. **o**, **p** Invasion assays of FaDu and Tu686 cells after exposed with CM from sCAFs or rCAFs. **q**, **r** Representative bright field images of tumor spheres from each group are given. Bar charts show the relative sphere area in each group (*n* = 3 independent samples). **s** Schematics diagram depicting the DDP treatment strategy of mice bearing tumors derived from subcutaneous injection of FaDu cells together with/without sCAFs or rCAFs at 1 to 3 ratio. **t** Representative gross tumor image from each treatment group is given. **u** Line graph shows the tumor growth of each group (*n* = 5 mice per group). **v** Bar chart shows the mean of tumor weight in each group. Data are shown as means ± S.E.M. *P < 0.05, **P < 0.01, ***P < 0.001, N.S. non-significant. **c**–**r**, **v** One-way ANOVA. **u** Two-way ANOVA. Scale bars in (**c**–**h, m**–**r**) represent 100 μm, (**t**) 1 cm.

To exploit the role of rCAFs in modulating cancer cell chemoresistance, we co-cultured HNSCC cancer cell lines (FaDu and Tu686) with sCAFs or rCAFs in a transwell system for 6 days, while the cancer cells were then treated with an escalated dose of 5’FU or DDP. Remarkably, IC_50_ experiments showed that the cancer cells co-cultured with rCAFs showed a significant increase in the DDP or 5’FU IC_50_ doses compared to those co-cultured with sCAFs or untreated cells (ctrl) (Fig. 2a, b and Supplementary Fig. 2g, h). This observations were further confirmed by cell counting experiments, which showed that the efficacy of chemotherapeutic drugs against cancer cell growth (GFP stably expressing cancer cells) was reduced when co-cultured with RFP (red fluorescent protein) stably expressing rCAFs compared with GFP expressing cancer cells co-cultured with RFP-labeled sCAFs (Fig. 2c, d and Supplementary Fig. 2i, j), while the results also showed that rCAFs were also less sensitive to chemotherapy treatment even in this co-culture experimental setting (Fig. 2c, d and Supplementary Fig. 2i, j). Furthermore, transwell migration and invasion assays indicated that co-culturing cancer cells with rCAFs enhanced their migration and invasion in the presence of 5’FU or DDP when compared to the cancer cells co-cultured with sCAFs or untreated cancer cells (Fig. 2e–h and Supplementary Fig. 2k–n). In contrast, our data showed that co-culturing cancer cells with sCAFs or rCAFs had no obvious effect on their growth, migration, and invasion ability in the absence of chemotherapeutic drugs as compared to untreated cancer cells (Fig. 2c–h, and Supplementary Fig. 2i–n).

To examine whether rCAFs contribute to cancer cell chemoresistance via paracrine effect, we exposed cancer cells to conditioned medium (CM) harvested from sCAFs and rCAFs, respectively. The results showed that exposing FaDu or Tu686 cells to CM from rCAFs increased their IC_50_ values for 5’FU or DDP and their colony formation ability in the presence of DDP or 5’FU compared to cells exposed to CM from sCAFs or untreated cancer cells (Fig. 2i–l, Supplementary Fig. 3a–d). Additionally, transwell migration and invasion assays showed that exposing FaDu and Tu686 cells to CM from rCAFs enhanced their migration and invasion abilities in the presence of 5’FU or DDP compared to cells exposed with CM from sCAFs or untreated cancer cells (Fig. 2m–p, Supplementary Fig. 3e–h). Notably, in the absence of chemotherapeutic drugs, exposing cancer cells to CM from rCAFs or sCAFs had a minimal effect on their colony formation, migration, and invasion abilities compared to untreated cancer cells (Fig. 2k–r, Supplementary Fig. 3c–h). Tumor sphere formation assays indicated that co-culturing cancer cells with rCAFs significantly increased their tumor sphere formation compared to cells co-cultured with sCAFs or untreated cells (Fig. 2q). Similarly, exposing cancer cells to CM from rCAFs also increased their tumor sphere formation (Fig. 2r). Next, we attempted to assess the effect of rCAFs on cancer cell chemosensitivity in vivo. Strikingly, our results showed that treatment with DDP was less effective in inhibiting the growth of tumor derived from subcutaneous co-injection of rCAF and FaDu cells compared to tumors derived from either subcutaneous co-injection of FaDu cells and sCAFs or injection of FaDu cells alone (Fig. 2s–v), indicating that rCAFs can confer cancer chemoresistance in vitro and in vivo.

To further confirm the paracrine nature of rCAFs mediated cancer cell chemoresistance, we performed heat inactivation pre-treatment with CM collected from rCAFs, which were then used for IC_50_ experiments in cancer cells. Our data indicated that exposure of cancer cells to pre-heated CM from rCAFs reduced their DDP or 5’FU IC_50_ values as compared to the cells exposed to untreated CM from rCAFs (Supplementary Fig. 3i–l). Collectively, these data suggested that NACT-resistant and -sensitive HNSCC patient derived tumors contain functionally diverse CAFs with different chemosensitivity, while rCAFs might modulate cancer cell chemoresistance through a paracrine effect.

### Comparison of sCAFs and rCAFs using multi-omics analysis reveals enriched pathways for cell survival and cytokine secretion in rCAFs

**Fig. 3:**
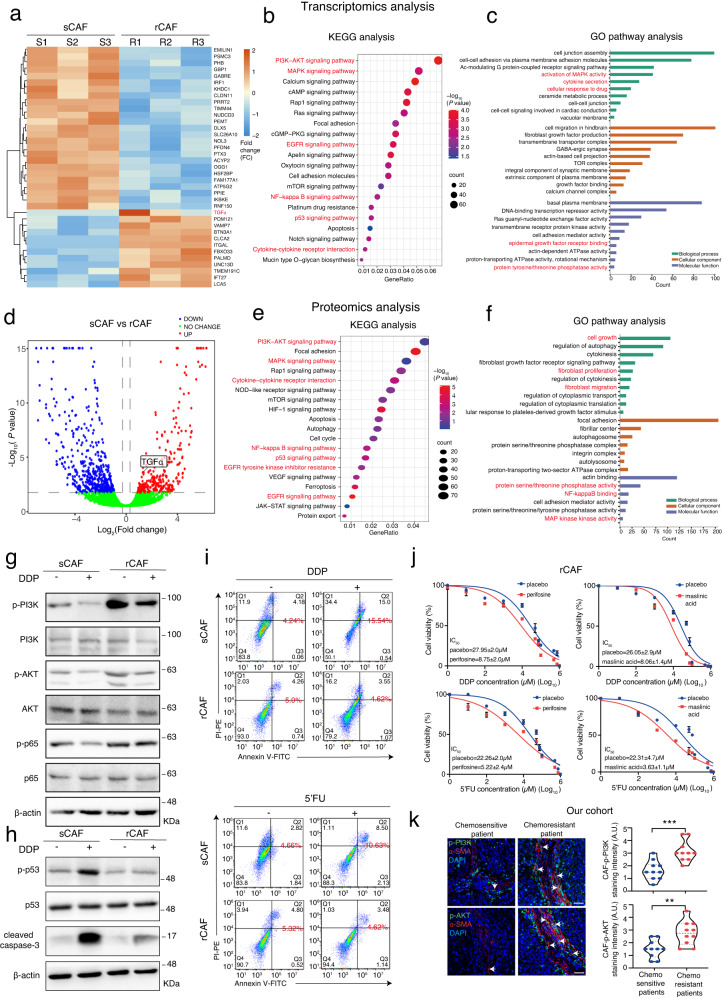
Multi-omics analysis reveals significant enrichment in some important signaling pathways and cellular processes in rCAFs compared to sCAFs. **a** Heatmap analysis of the transcriptomics data from three different NACT-sensitive and -resistant HNSCC patients derived CAFs respectively. **b**, **c** KEGG and GO analysis of the transcriptomics data between sCAFs and rCAFs. Red color highlights the signaling, cellular and biological pathways and molecular functions related to this study. **d** Volcano plot showing the comparative proteomics profiling analysis of three different NACT-sensitive and -resistant HNSCC patients derived CAFs. **e**, **f** KEGG and GO analysis of the proteomics data from three different sCAFs and rCAFs respectively. Red color highlights the signaling, cellular and biological pathways and molecular functions involved in this study. **g**, **h** Western blot analysis of the indicated proteins in sCAFs and rCAFs in the presence or absence of 8 μM DDP. Molecular markers (kDa) are shown on the right-hand side. **i** Annexin V-PI apoptotic assays of rCAFs and sCAFs after treated with or without 8 μM DDP or 15 μM 5’FU. The percentage of viable cells, early apoptotic cells, and late apoptotic cells was determined based on the lower quadrant (Q4), lower right quadrant (Q3), and upper quadrant (Q2), respectively. The red number indicates the percentage of apoptotic cells in each group. **j** DDP or 5’FU IC_50_ experiments of sCAFs and rCAFs after treated with or without 10 μM AKT (Perifosine) or 20 μM p65 inhibitors (Maslinic acid) (*n* = 3 independent experiments). **k** Representative co-immunofluorescent staining of p-P13K/p-AKT and α-SMA in tumor sections from each group. Bar charts represents the staining intensity in each group as indicated (*n* = 20 patients, our cohort). Arrow indicates p-P13K or p-AKT and α-SMA double positive cells. Data are shown as means ± S.E.M. **P < 0.01, ***P < 0.001. **a**–**f**, **k** Student’s *t* test. Scale bars in (**k**) represent 100 μm.

To study the molecular mechanism behind these functionally distinct CAFs, we conducted transcriptomics analysis using sCAFs and rCAFs isolated from three NACT-sensitive and -resistant HNSCC patients, respectively. Heatmap analysis of the transcriptomics data indicated a significant different gene expression signature between sCAFs and rCAFs (Fig. 3a), while no difference was observed in the expression of conventional fibroblast markers, including *ACTA2, FAP* and *PDGFA*, between them (Supplementary Fig. 4a). KEGG and GO analysis of the transcriptomics data indicated that, compared to sCAFs, rCAFs were significantly enriched in signaling pathways and cellular processes such as PI3K/AKT, MAPK, EGFR (epidermal growth factor receptor), NF-kB and p53 signaling pathways, cell growth, cellular response to drug, cytokine secretion, cytokine-cytokine receptor interaction, among others (Fig. 3b, c). To confirm our findings, we carried out proteomics analysis of the sCAFs and rCAFs. Similar to the transcriptomics data, KEGG and GO analysis of the proteomics data indicated significant enrichment in PI3K/AKT, NF-kB, p53 or EGFR signaling pathway, as well as cytokine-cytokine receptor interaction, fibroblast proliferation and cell growth in rCAFs compared to sCAFs (Fig. 3d–f). As PI3K/AKT, NF-kB or p53 signaling pathway were known to regulate cell proliferation, cytokine secretion and apoptosis^9,25,26^, we examined whether these pathways contributed to the reduced chemosensitivity observed in rCAFs. Western blot analysis showed that treatment with DPP or 5’FU reduced phosphorylation levels of PI3K, AKT, and NF-kBp65 in sCAFs as compared with placebo-treated cells. While these treatments exhibited a more pronounced effect on reducing the phosphorylation levels of PI3K, AKT, and NF-kBp65 in sCAFs, there was also a modest reduction in p-PI3K, p-AKT and p-p65 levels in rCAFs (Fig. 3g, Supplementary Fig. 4b). In contrast, the level of p-p53 and caspase-3 cleavage was dramatically increased in sCAFs but not in rCAFs after treatment with 5’FU and DDP compared to placebo treated cells (Fig. 3h, Supplementary Fig. 4c). Interestingly, our results indicated that AKT inhibitor treatment reduced the phosphorylation of p65 in rCAFs (Supplementary Fig. 4d), indicating that its mediated p65 activation in rCAFs. Apoptotic assays also showed that 5’FU or DDP treatment increased apoptosis in sCAFs but not in rCAFs (Fig. 3i) Treatment with PI3K or AKT inhibitor reduced the 5’FU or DDP IC_50_ value of rCAFs and increased apoptosis in rCAFs after treated with chemotherapeutic drugs (Fig. 3j, Supplementary Fig. 4e, f). Consistent with this finding, previous studies showed that elevated PI3K/AKT signaling pathway activity can inhibit chemotherapy-induced p53/caspase-3 dependent apoptosis in cancer cells^27,28^.

We next assessed the clinical significance of our observation by performing co-immunostaining of p-PI3K, p-AKT or Ki67 and α-SMA in tissue sections derived from chemo-sensitive and -resistant patients, respectively, indicating that the expression of p-PI3K and p-AKT was up-regulated in rCAFs as compared to sCAFs (Fig. 3k), while the relative number of Ki67 positive CAFs was higher in chemoresistant patients derived tumors than in those from chemosensitive patients (Supplementary Fig. 4g). Overall, our results provide the first explanation for the observed unchanged CAF population in chemoresistant tumors before and after the NACT treatment, suggesting that the elevated PI3K/AKT signaling pathway in rCAFs may have protected them against chemotherapy-induced apoptosis.

### Crosstalk between rCAFs and cancer cells promotes cancer chemoresistance via TGFα-EGFR paracrine signaling

**Fig. 4:**
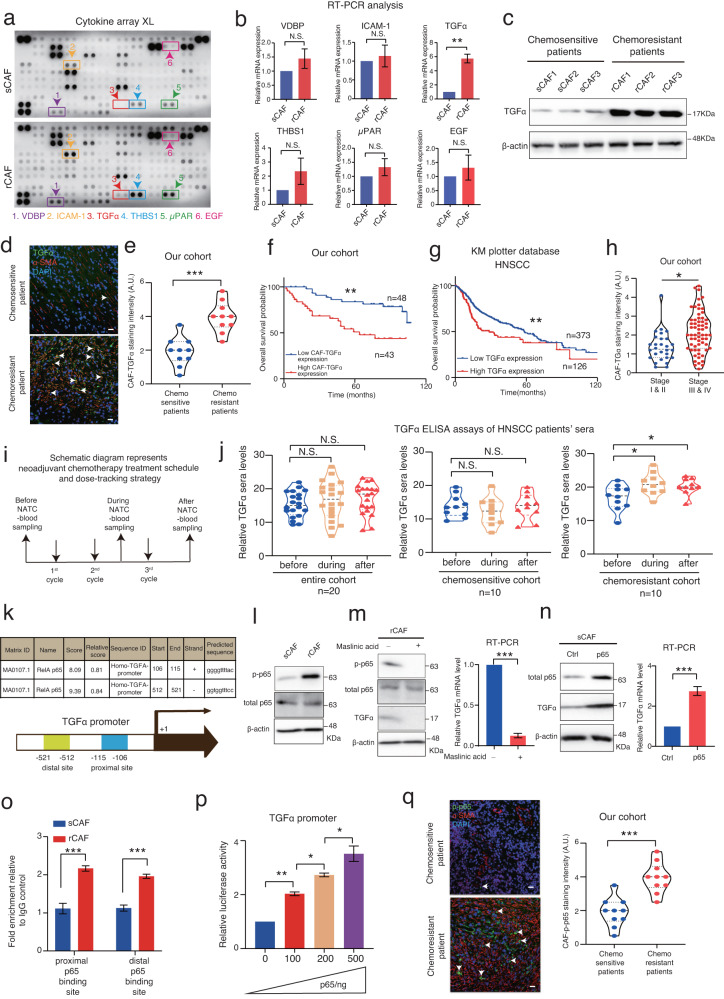
The level of TGFα secreted by rCAFs is elevated and correlates with chemoresistance in HNSCC patients. **a** Cytokine XL array analysis of sCAFs and rCAFs. Boxes indicate the selected cytokines in rCAFs. **b**, **c** RT-PCR and western blot analysis of the indicated cytokines in rCAFs relative to sCAFs (*n* = 3 chemo-sensitive or chemo-resistant patients). **d** Co-immunofluorescent staining of TGFα and α-SMA expression in tumor sections from chemo-sensitive and -resistant patients respectively. Arrow indicate TGFα and α-SMA double positive cells. **e** Violin plot showing the mean staining intensity of CAF-TGFα in each group (*n* = 20 patients, our cohort). A.U. stands for arbitrary unit. **f** Kaplan–Meier survival study of the HNSCC patients with high or low CAF-TGFα expression (*n* = 91 patients, our cohort). **g** The relationship between TGFα expression and overall survival in HNSCC patients (*n* = 499 patients, KM plotter database). **h** The corelation between CAF-TGFα expression and cancer progression in HNSCC patients (*n* = 91 patients, our cohort). **i** Schematics diagram represents the blood sampling strategy of HNSCC patients who received NACT treatment. **j** Violin plots show the level of serum TGF in entire cohort, chemosensitive or chemoresistant patients before, during and after receiving NACT treatment. **k** Table shows the putative p65 binding sites on human TGFα promoter. **l** Western blot analysis of the expression of p-p65 and total p65 in sCAFs and rCAFs. β-actin was used as a loading control. **m** Western blot and RT-PCR analysis of rCAFs after treated with or without 20 μM Maslinic acid as indicated. **n** Western blot and RT-PCR analysis of sCAFs transfected with p65 overexpression vector or empty vector (ctrl). **o** ChIP assays. Bar charts show the relative enrichment of p65 binding on TGFα promoter in rCAFs as compared to sCAFs (*n* = 3 independent experiments). **p** Luciferase assay of sCAFs after co-transfected with a luciferase reporter vector containing TGFα promoter and an escalated amount of p65 overexpression plasmid (*n* = 3 independent experiments). **q** Co-immunofluorescent staining of p-p65 and α-SMA in tumor sections from each group (*n* = 20 patients, our cohort). Arrow indicates p-p65 and α-SMA double positive cells. Data are shown as means ± S.E.M. *P < 0.05, **P < 0.01, ***P < 0.001, N.S. non-significant. **b**, **e**, **h**, **q** Student’s *t* test. **f**, **g** Log-rank (Mantel-Cox) test. **j**, **o**, **p** One-way ANOVA. Scale bars in (**d**, **q**) represent 100 μm.

We next sought to dissect the underlying molecular mechanism of rCAF mediated paracrine effect on cancer cell chemoresistance. By performing a proteome profiler human XL cytokine array with sCAFs and rCAFs, we showed that the expression of TGFα (tumor growth factor alpha), vitamin D binding protein, ICAM-1 (intracellular adhesion molecule 1), THBS1 (Thrombospondin 1) and μPAR (urokinase plasminogen activator receptor), among others, was up-regulated in rCAFs as compared to sCAFs (Fig. 4a). The results of RT-PCR analysis demonstrated that TGF-α exhibited consistent up-regulation in CAFs derived from three representative chemoresistant HNSCC patients compared to sCAFs, as evidenced by the western blot analysis (Fig. 4b, c). Consistent with this finding, TGFα, as the main ligand for EGFR, has been linked to patient prognosis in different cancers, including HNSCC^29,30^, whereas its regulatory role in chemotherapeutic response was largely unknown. Notably, EGF (epidermal growth factor) did not show any significant change between sCAFs and rCAFs (Fig. 4a, b). To demonstrate the clinical relevance of our findings, we co-immunostained tumor tissue sections derived from chemo-sensitive and -resistant patients with TGFα and α-SMA antibodies. Our results showed that the expression of CAF-TGFα was up-regulated in chemoresistant patient-derived tumors as compared with chemosensitive ones, and its expression was predominately expressed in rCAFs within the tumor microenvironment (Fig. 4d, e). Importantly, we also showed that higher expression of CAF-TGFα or tumor-TGFα defined worse overall survival and escalated cancer stage progression in HNSCC patients (Fig. 4f–h). To evaluate the potential diagnostic value of TGFα expression, we also conducted a drug response tracking experiment in which we collected sera from HNSCC patients before, during, and after they received NACT treatment, which were then subjected to ELISA analysis (Fig. 4i). Our results indicated that the serum level of TGFα was elevated in chemoresistant patients during and after the NACT treatment but not in chemosensitive patients or the entire cohort of HNSCC patients (Fig. 4j). Notably, the serum level of TGFα was also significantly higher in chemoresistant patients compared to chemosensitive patients before receiving the NACT treatment (Supplementary Fig 5a). These findings suggest that the elevated serum TGFα level could predict the onset of chemoresistance in HNSCC patients.

Both the transcriptomics and proteomics analysis highlighted elevated PI3K/AKT/p65 signaling pathways in rCAFs. Given that p65 is known to regulate cytokine production in various cell types^26^, we investigated whether it plays a role in TGFα secretion by rCAFs. To this end, we performed a transcriptional factor binding site prediction analysis of the TGFα promoter, which revealed 2 putative p65 binding sites (Fig. 4k). Our data indicated that p65 phosphorylation levels were higher in rCAFs than in sCAFs (Fig. 4l), and treatment with a p65 inhibitor reduced TGFα expression in rCAFs compared with placebo-treated rCAFs (Fig. 4m). Additionally, overexpression of p65 in sCAFs enhanced TGFα expression compared to sCAFs transfected with an empty vector (Fig. 4n). We then performed ChIP (chromatin immunoprecipitation) experiments with a p65 antibody, which showed increased p65 occupancy on the putative binding sites of TGFα promoter in rCAFs compared with sCAFs (Fig. 4o). Furthermore, a luciferase assay revealed dose-dependent activation of TGFα promoter activity in sCAFs co-transfected with a luciferase reporter containing the full-length wild-type TGFα promoter sequence and increasing amounts of the p65 overexpression vector (Fig. 4p). Clinically, co-immunostaining analysis of α-SMA and p-p65 expression showed increased levels of CAF-p-p65 expression in chemoresistant patient-derived tumors compared to chemosensitive patient-derived tumors (Fig. 4q). Overall, our results suggest that p65 acts as an upstream regulator of TGFα secretion in rCAFs, thereby modulating the crosstalk between cancer cells and rCAFs.

### Suppression of TGFα in rCAFs represses their paracrine influence on cancer cell chemoresistance

**Fig. 5:**
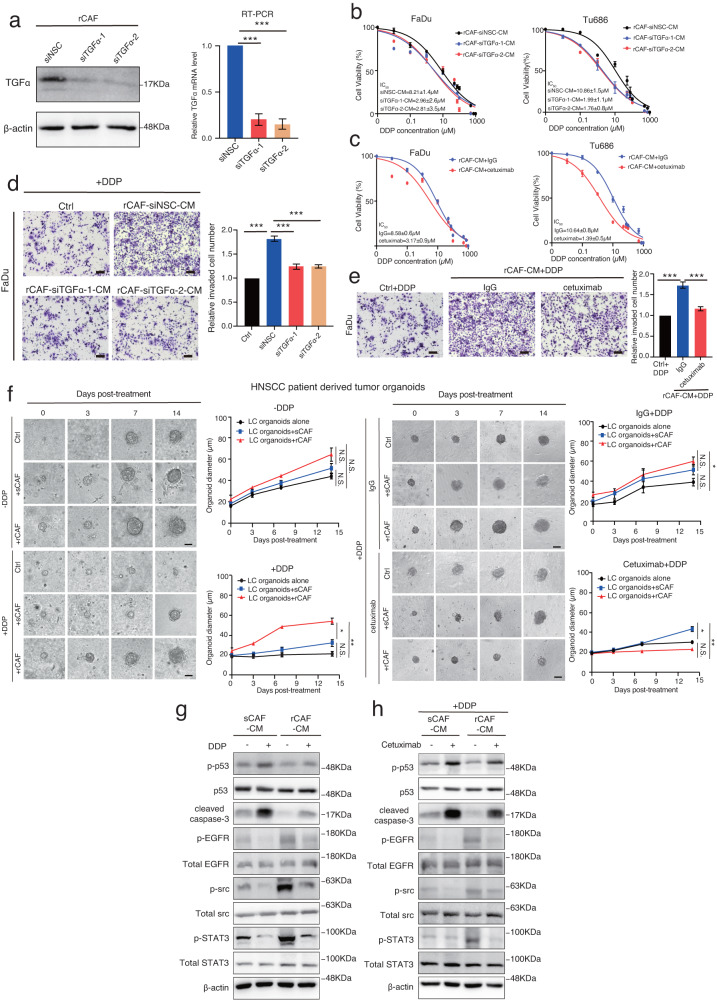
Depletion of TGFα rescues the promoting effect of rCAFs on chemoresistance in both tumor cells and patient derived organoids. **a** Western blot and RT-PCR analysis of the TGFα expression in rCAFs after transfected with non-silencing control (NSC) siRNA or TGFα targeting siRNA-1/-2. **b** DDP IC_50_ experiments of FaDu or Tu686 cells after exposed with either CM from siTGFα or siNSC transfected rCAFs. **c** DDP IC_50_ experiments of FaDu or Tu686 cells after treated with CM from rCAFs in the presence of IgG control or cetuximab (*n* = 3 independent experiments). **d** Transwell invasion assays of DDP treated cancer cells in the presence of CM from siTGFα or siNSC transfected rCAFs. **e** Invasion assays for DDP treated FaDu cells exposed with CM from rCAFs in the presence or absence of IgG antibody or cetuximab. Bar charts show the relative invaded cell number in each group (*n* = 3 independent experiments). **f** Representative bright field images of HNSCC patient derived organoids after co-culture with/without rCAFs or sCAFs in the presence or absence of DDP -/+ IgG control or cetuximab. Line graphs show the growth of tumor organoids in each treatment group (*n* = 3 independent experiments). Ctrl represents tumor organoids without co-culture with sCAF or rCAF. **g** Western blot analysis of p-EGFR, total EGFR, p-Src, total Src, p-STAT3, and total STAT3 expression in FaDu cells after treated with CM from sCAFs or rCAFs in the presence or absence of 3 μM DDP. **h** Western blot analysis of the indicated protein expression in FaDu cells after treated with 3 μM DDP in the presence of CM from sCAFs or rCAFs together with or without 100 μg/mL cetuximab. Data are shown as means ± S.E.M. *P < 0.05, **P < 0.01, ***P < 0.001, N.S. not significant. **a**, **d**, **e** One-way ANOVA. **f** Two-way ANOVA. Scale bars in (**d**, **e**) represent 100 μm, (**f**) 200 μm.

To examine the effect of TGFα expression on rCAF mediated cancer cell chemoresistance, we next transfected rCAFs with TGFα targeting siRNAs, while the knock-down effect of TGFα production was confirmed by western blot and RT-PCR analyses (Fig. 5a). Strikingly, exposure of FaDu or Tu686 cells with either CM from TGFα depleted rCAFs or CM from rCAFs supplemented with anti-EGFR cetuximab reduced their 5’FU or DDP IC_50_ values, as well as colony formation ability in the presence of DDP or 5’FU, compared with the cells exposed to either CM from rCAFs transfected with non-silencing control siRNA (siNSC) or CM from rCAFs supplemented with IgG antibody, and untreated control cells (Fig. 5b, c, Supplementary Fig. 5b–i). Interestingly, exposure of cancer cells to CM harvested from p65 inhibitor pre-treated rCAFs also reduced their colony formation in the presence of DDP or 5’FU, compared with the cells treated with placebo pre-treated rCAFs (Supplementary Fig. 5g–i), whereas it had no obvious effect on the colony formation in the cancer cells exposed with either CM from p65 or TGFα-depleted rCAFs or siNSC transfected rCAFs in the absence of chemotherapeutic drugs (Supplementary Fig. 5d–i). Furthermore, transwell invasion assays indicated that exposure of cancer cells to either CM from TGFα depleted rCAFs or CM from CAFs supplemented with cetuximab reduced their invasion ability in the presence of DDP, compared with the cells exposed to either CM from siNSC transfected rCAFs or CM from rCAFs supplemented with IgG, and untreated control cells (Fig. 5d, e). To test the clinical relevance of our findings, we developed a method to generate HNSCC patient tumor-derived organoids, which were then used to examine whether treatment with cetuximab would prohibit the paracrine effect of rCAFs on chemosensitivity in a tumor organoid experimental setting. Strikingly, our results indicated that co-culturing of HNSCC tumor-derived organoids with rCAFs enhanced their growth in the presence of DDP, compared with the tumor organoids co-cultured with sCAFs or tumor organoids alone group, while treatment of cetuximab reduced the enhanced growth observed in tumor organoids co-cultured with rCAFs, compared with IgG treated group (Fig. 5f). To further confirm whether rCAFs regulated cancer cell chemoresistance via EGFR signaling pathway, we performed western blot analysis with cancer cells after treated with CM from sCAFs or rCAFs in the presence or absence of DDP or 5’FU, indicating that the phosphorylation of EGFR and its downstream effectors Src and STAT3^31,32^ was increased in FaDu or Tu686 cells after exposed with CM from rCAFs even in the presence or absence of chemotherapy, compared to the cells exposed with CM from sCAFs (Fig. 5g, Supplementary Fig. 5j, k). Moreover, cetuximab treatment reduced the enhanced EGFR, Src or STAT3 phosphorylation observed in chemotherapy treated FaDu or Tu686 cells after treatment with CM from rCAFs (Fig. 5h, Supplementary Fig. 5l and m). In addition, the phosphorylation of p53 and caspase-3 cleavage was decreased in cancer cells after exposure to CM from rCAFs, even in the presence of DDP or 5’FU, compared to the cells treated with CM from sCAFs (Fig. 5g, Supplementary Fig. 5j, k). Administration of cetuximab, however, rescued the DDP or 5’FU-induced p53 phosphorylation and caspase-3 cleavage in both Fadu and Tu686 cancer cell lines after exposure to CM from rCAFs (Fig. 5h, Supplementary Fig. 5l and m). Overall, our results indicate that rCAFs mediate cancer cell chemoresistance via the TGFα-EGFR paracrine signaling.

### Cetuximab treatment rescues the chemosensitivity of tumors derived from co-injection of HNSCC cells and rCAFs

**Fig. 6:**
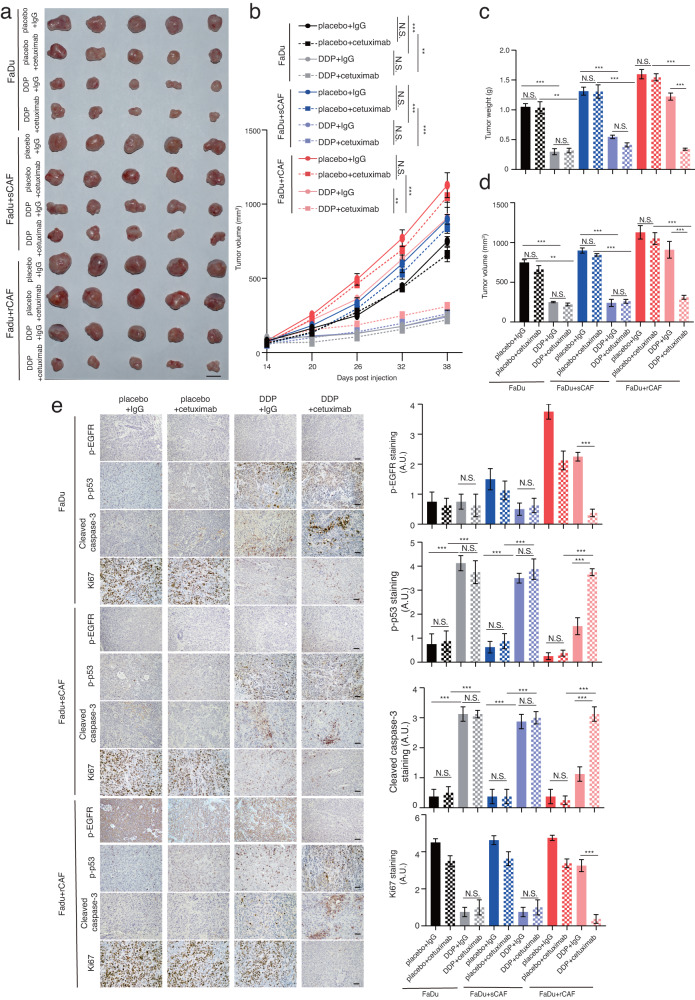
Administration of cetuximab rescues cisplatin efficacy against tumor growth in mice that were subcutaneously co-injected with FaDu cells and rCAFs. **a** Representative gross tumor image from each treatment group is given. **b** Line graphs show the tumor growth in each treatment group (*n* = 5 mice per group). Bar chart represent the mean of final tumor weight (**c**) or volume (**d**) in each group. Data are shown as means ± S.E.M. **e** IHC analysis of p-EGFR, p-p53, cleaved caspase-3 and Ki67 staining in tumor sections derived from each treatment group (*n* = 5 tumors analyzed per group). Bar charts represent the quantification of staining intensity in each group. Data are shown as means ± S.E.M. *P < 0.05, **P < 0.01, ***P < 0.001, N.S. non-significant. **b** Two-way ANOVA. **c**–**e** One-way ANOVA. Scale bars in (**a**) represent 1 cm, (**e**) 100 μm.

To examine the regulatory role of rCAFs in cancer cell chemoresistance in vivo, we conducted subcutaneous co-injection of FaDu cells with or without sCAFs or rCAFs (at a 1 to 3 ratio: cancer cell to CAFs) into nude mice, which were then treated with either placebo, DDP, DDP with IgG antibody or DDP with cetuximab. Our results indicated that co-administration of cetuximab and DDP significantly inhibited tumor growth in mice that were co-injected with FaDu cells and rCAFs compared to the mice that were treated with either placebo, DDP alone or the combination of IgG antibody and DDP, whereas it had no synergic effect against tumor growth in mice that were co-injected with FaDu cells and sCAFs or FaDu cells alone (Fig. 6a–d). In contrast, cetuximab treatment alone had a modest effect on tumor growth in mice that were co-injected with FaDu cells and sCAFs or rCAFs, or injected with FaDu cells alone (Fig. 6a–d). Importantly, immunofluorescent staining experiment revealed an increased CAF population in the tumors derived from the co-injection of FaDu cells with sCAFs or rCAFs and treated with placebo and IgG, in comparison to tumors derived from the injection of FaDu cells alone (Supplementary Fig. 6), suggesting the injected sCAFs or rCAFs were highly likely retained in the tumors. Further IHC examination indicated that the phosphorylation level of EGFR and Ki67 staining were increased in tumors-derived from co-injection of FaDu cells and rCAFs, even in the presence of DDP treatment, compared to tumors arising from either FaDu cell and sCAF co-injection or injection of FaDu cells alone (Fig. 6e). Notably, the increased staining intensity of p-EGFR and Ki67 observed in the FaDu and rCAF co-injected tumors was significantly reduced after treatment with cetuximab and DDP when compared with IgG control antibody and DDP-treated group (Fig. 6e). As EGFR pathway activation has been shown to inhibit p53/caspase-3 dependent cell apoptosis^33^, our data also indicated that the tumor expression of p-p53 and caspase-3 cleavage was decreased in DDP treated mice that were co-injected with FaDu cells and rCAFs compared with the DDP treated mice that were either co-injected with FaDu and sCAFs or injected with FaDu cells only (Fig. 6e). However, cetuximab treatment rescued chemotherapy-induced p-p53 phosphorylation and caspase-3 cleavage in the FaDu cell and rCAF co-injected tumors compared to the tumors treated with IgG control antibody and DDP (Fig. 6e).

**Fig. 7:**
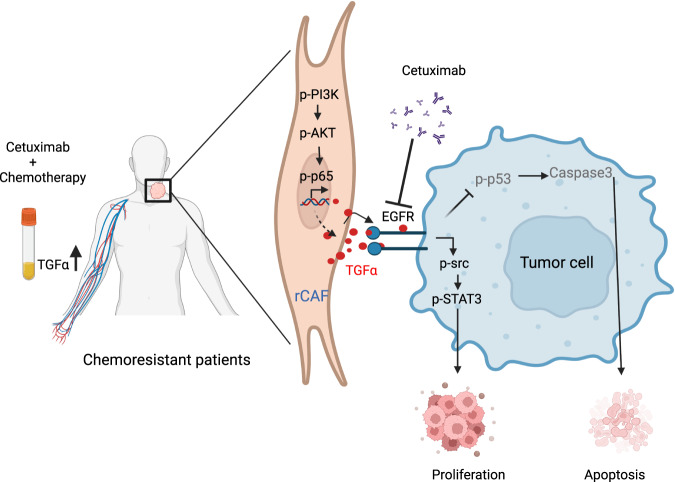
Schematic diagram represents the regulatory role of tumor cell-rCAF crosstalk in chemoresistance. In chemoresistant HNSCC patients, the population of CAFs in tumors remains unchanged before and after receiving NACT treatment. Mechanistically, the elevated PI3K/AKT/p65 signaling pathway in rCAFs prohibits chemotherapy-induced cell death and up-regulates TGFα secretion. rCAF-secreted TGFα then binds and activates its receptor EGFR in cancer cells, which subsequently up-regulates its downstream Src/STAT3 mediated survival pathway and represses p53/caspase-3 dependent apoptosis to cause tumor chemoresistance. Clinically, the elevated TGFα serum level determines NACT response in patients with HNSCC, while treatment with clinically approved anti-EGFR cetuximab can rescue chemotherapeutic response in tumors that are derived from co-injection of cancer cells and rCAFs in vivo.

Overall, our works uncover new insights whereby the increased CAF population correlates with worse chemotherapeutic responses and poor prognosis in HNSCC patients, while the number of CAFs remains unchanged in chemoresistant patient derived tumors before and after receiving NACT treatment. Mechanistically, we show that the elevated PI3K/AKT/p65 signaling axis in rCAFs prohibits chemotherapy-induced cell death and increases TGFα secretion, which, in turn, promotes cancer cell chemoresistance by activating the EGFR/Src/STAT3 mediated cell survival pathway and repressing the p53/caspase-3 dependent apoptosis. Importantly, a dose-response tracking experiment reveals an elevated TGFα serum level in chemoresistant HNSCC patients during and after receiving NACT treatment, while administration of anti-EGFR cetuximab rescues chemotherapeutic response in tumors derived from the co-injection of cancer cells and rCAFs in vivo. These findings identify a new serum marker and potent combined therapy for chemoresistant HNSCC patients (Fig. 7).

## Discussion

Our work presents a highly effective method to isolate CAFs from pre-treatment tumor biopsies of NACT-sensitive and -resistant HNSCC patients. Through multi-omics analyses of primary CAFs, we reveal an elevated PI3K/AKT/p65 mediated cell survival and cytokine production in rCAFs compared to sCAFs. This contributes to the increased CAF population in chemoresistant patient-derived tumors compared to chemosensitive patient-derived tumors and promotes cancer cell chemoresistance through TGFα-EGFR paracrine signaling. In vivo, co-injection of cancer cells and rCAFs reduces cisplatin efficacy against tumor growth compared to tumors derived from either co-injection of sCAFs and cancer cells or cancer cells alone. However, treatment with cetuximab rescues the chemotherapeutic response of cancer cells and rCAF co-injected tumors. Our results indicate that disrupting the tumor cell-rCAF crosstalk can potentially enhance chemotherapy efficacy in HNSCC patients.

Although HNSCC patients routinely receive with chemotherapy, many of them are poorly responsive or resistant to the treatment^3,7^. Previous studies have focused on exploring how tumor cells escape chemotherapy-induced cell death^34^, while the role of stromal cells, particular their dominant player-CAFs, in HNSCC chemoresistance is largely unknown. Cancer associated fibroblasts have been shown to play a key regulatory role in tumor cell growth, survival and metastasis via either paracrine effect or cell-cell contract^14,15,35^. Nevertheless, their role in HNSCC chemoresistance has not been explored until this study. Our clinical study shows that the tumor-CAF population significantly decreases in chemosensitive patients after receiving NACT treatment, while there is no significant change in CAF population between the residual tumors and tumor samples obtained prior to treatment from chemoresistant patients. To exploit the underlying mechanism behind this difference, we developed an effective method to isolate and purify CAFs from chemo-sensitive and -resistant HNSCC patients, respectively. Our characterization experiments indicate no significant difference in basic cell properties between sCAFs and rCAFs, whereas functional studies reveal that rCAFs are more resistant to 5’FU or DDP-induced cell death compared to sCAFs. Clinically, the relative number of Ki67 positive CAFs is increased in chemoresistant patient-derived tumors compared to chemosensitive patients. Thus, these results probably explain why the population of tumor-CAF in chemoresistant patients remains unchanged before and after NACT treatment. Consistently, several studies have suggested that chemotherapeutic drugs may influence the survival and properties of stromal cells^16,17^. On the other hand, previous studies have shown that CAFs are highly heterogenous and composed of different CAF subsets that exert a wide range of distinct functions on tumor cells^36^. Herein, we show that co-culturing tumor cells with rCAFs or exposing cancer cells to CM from rCAFs enhances their resistance to chemotherapeutic drugs compared to tumor cells either co-cultured with sCAF or exposed to CM derived from sCAFs, while co-injection of cancer cells and rCAFs reduces cisplatin efficacy against tumor growth in vivo. Furthermore, we show that exposure of cancer cells with heat inactivated CM from rCAFs can no longer affect chemosensitivity in cancer cells, suggesting the paracrine nature of rCAF mediated cancer cell chemoresistance.

We next explored the underlying mechanism of rCAF-mediated cancer cell chemoresistance by performing combined transcriptomics and proteomics analyses with sCAFs and rCAFs. Our findings indicated an elevated PI3K/AKT/NF-κB p65 signaling pathways and cytokine secretion in rCAFs as compared to sCAFs. Indeed, previous studies show that activation of the PI3K/AKT signaling pathway can regulate NF-κB p65 transcriptional activity to modulate cell survival and cytokine production^37,38^. Our biochemical analyses indicate a significant alteration in cytokine production within rCAFs. Among these cytokines, TGFα is the only cytokine that consistently up-regulated in three different HNSCC patient derived rCAFs, while EGF is not. Depletion of TGFα prohibits rCAF mediated chemoresistance in cancer cells. Previous studies have demonstrated that TGFα is a more potent agonist for EGFR than EGF in various biological systems^23,24^, highlighting the significance of CAF-derived TGFα in regulating EGFR-mediated cancer cell chemoresistance. Additionally, we first show that p65 can regulate TGFα production transcriptionally by binding its promoter directly. Clinically, our data indicate that in HNSCC patients, the levels of p-PI3K, p-AKT, p-p65 and TGFα in CAFs is up-regulated in chemoresistant derived tumors as compared to chemosensitive derived tumors, while TGFα is predominately expressed in rCAFs and its expression correlates with patient prognosis. In agreement with this finding, previous studies have reported that TGFα plays an important role in regulating cancer cell survival by interacting with its receptor EGFR^23^, which then activate its downstream Src/STAT3 mediated cell survival pathway to prohibit p53/caspase-3 driven apoptosis^31–33^. EGFR expression is known to be frequently overexpressed in HNSCC, probably due to high frequency of EGFR amplification^39^. Indeed, our mechanistic study also shows that rCAF-secreted TGFα activates the EGFR/Src/STAT3 signaling pathway in cancer cells, which in turn increases cell survival and prohibits p53/caspase-3 dependent apoptosis during chemotherapy treatment. Collectively, our results suggest that in chemoresistant HNSCC patients, rCAF is the main supplier of TGFα in the tumor microenvironment, which may affect cancer cell chemoresistance via the TGFα-EGFR paracrine signaling.

Cetuximab is the only anti-EGFR drug that has been approved for treating HNSCC^40^, since EGFR is predominantly expressed in HNSCC tumors. In the past, cetuximab was frequently given in combination with radiotherapy to HNSCC patients who unfitted to receive high dose cisplatin or those patients had already received several cycles of cisplatin-based NACT with severe side effect^6,41^. In the EXTREME trial, co-administration of cetuximab and first-line chemotherapy has been shown to improve disease control and increase overall survival in advanced HNSCC patients when compared to chemotherapy alone^21^. Nevertheless, its underlying mechanism and therapeutic response predictor has not been fully exploited until this study. We show that treatment with cetuximab or TGFα depletion represses the promoting effect of rCAFs on chemoresistance in both cancer cell lines and patient derived organoids. Using a murine HNSCC model, our results indicate that treatment with cetuximab rescues the chemosensitivity of tumors in mice that were co-injected with rCAFs and FaDu cells as compared to IgG control antibody and cisplatin treated group, while the serum level of TGFα is only escalated in chemoresistant HNSCC patients during and after the NACT treatment. In summary, our results explain why the addition of cetuximab can improve chemotherapy efficacy against HNSCC tumor growth because of its effect on disrupting tumor cell-CAF crosstalk. Additionally, we provide the first clinical evidence that the serum level of TGFα can predict patient response to NACT and warrants further clinical investigation.

Overall, our results uncover a previously unrecognized mechanism whereby the number of CAFs remains unchanged in chemoresistant HNSCC patients before and after receiving NACT treatment, primarily due to their resistance to chemotherapy-induced cell death. At the molecular level, rCAFs up-regulate PI3K/AKT/p65 driven cell survival pathway and TGFα production, which in turn protect cancer cells from chemotherapy-induced cell death by activating the EGFR/Src/STAT3 survival pathway and repressing the p53/caspase-3 driven apoptosis. These findings provide an improved treatment strategy for chemoresistant HNSCC patients.

## Method

### Clinical tissue sample preparation and collection

For the Kaplan-Meier survival study, HNSCC specimens were collected from patients who underwent surgery at the Sun Yat-sen Memorial Hospital with complete clinicopathological records. For the chemosensitivity study, we collected another cohort of the tumor biopsies and blood samples from HNSCC patients who received 3 cycles of cisplatin, 5’FU plus docetaxel based NACT. For drug response tracking experiments, the blood samples were collected from HNSCC patients before, during and after they received NACT treatment. Their response to chemotherapeutic agents was monitored by performing MRI before and after the NACT treatment: chemoresistant HNSCC patients were those with PD or SD, while the patients with complete response or partial response were classified as chemosensitive patients. For the IHC study of our HNSCC patient cohort, the median of α-SMA, PDGFRα, p-p65/α-SMA and TGFα/α-SMA staining was set as a cutoff threshold for stratifying patients into those with high or low expression group. All paraffin embedded/frozen samples were collected from HNSCC patients with written informed consent (as requested by the Declaration of Helsinki and Istanbul). The collection of clinical specimens and related procedures were carried out with the approval of the internal review and ethics committee of the Sun Yat-sen Memorial hospital (Ref no: SYSEC-KY-KS-2021-319).

### Online database analysis

The correlation study between the ratio of TGFα to fibroblast marker α-SMA expression and overall survival in cancer patients was done by using Kaplan–Meier (KM) plotter database online tool (http://kmplot.com/analysis/) according to the web tool developer’s instruction and described previously^42,43^.

### Isolation and purification of cancer associated fibroblasts from chemo-sensitive and -resistant patients

Fresh pre-treatment tumor biopsies were obtained from neoadjuvant chemosensitive or chemoresistant patients and rinsed with saline solution (0.9% NaCl) to remove blood. The specimens were micro-dissected into small pieces, each measuring less than 0.5 cm^3^. To prepare the tissues for analysis, enzymatic dissociation was performed at 37 °C on a shaking table for 45 min, using an incubation solution of 5 mL DMEM medium containing 1 mg/mL collagenase type 3 (Cat no.#LS004182, Worthington) and 1 mg/mL DNase I (Cat no.#10104159, Roche). The enzymatic digestion process was halted by adding 5 mL of DMEM medium with 10% FBS. The resulting cell suspension was then sequentially filtered through 100 μm and 70 μm strainers. Subsequently, the collected filtered cells were treated with 4 mL of RBC lysis buffer (Cat no.#420301, Biolegend) to remove red blood cells, and gently lysed for 2 min at room temperature. After centrifugation and resuspension in saline solution (0.9% NaCl), the cells underwent an additional round of centrifugation to eliminate any remaining red blood cells. The resulting pellet was resuspended in PBS containing 1% FBS and incubated with PE-conjugated anti-human FAP (Cat no.#FAB3715P, BioTechne) for 30 min at 4 °C. Magnetic activated cell sorting using anti-PE MicroBeads (Cat no.#130-048-801, Miltenyi Biotec) was then performed according to the manufacturer’s instructions. Finally, the sorted cells were cultured in DMEM medium supplemented with 10% fetal bovine serum and 1% penicillin and streptomycin, and seeded into 6-well plates, where they were left undisturbed for 5–7 days. Subsequently, the cells were expanded and subjected to FCM, coverslip immunofluorescent staining, and RT-PCR analyses to confirm their fibroblast identity. The FACS gating strategies were given in Supplementary Fig. 8a.

### Characterization of cancer associated fibroblasts

Primary fibroblasts were immunostained with fluorochrome-conjugated antibodies of the FACS step as described previously^43^. Briefly, cells were incubated with antibodies directed at endothelial marker BV421-conjugated anti-human CD34 (Cat no.#343610, Biolegend (1 in 100 dilution)) and PE-conjugated anti-human CD31 (Cat no.#343610, Biolegend, (1 in 100 dilution)), immune cell marker PE-conjugated anti-human CD45 (Cat no.#301706, Biolegend, (1 in 300 dilution)), fibroblast surface marker PE-conjugated anti-human FAP (Cat no.#FAB3715P, BioTechne, (1 in 300 dilution) and PE-conjugated anti-human PDGFA (Cat no.#323505, Biolegend, (1 in 100 dilution)) at 37 °C for 15 min. Finally, cells were washed and resuspended in PBS and analyzed by FCM.

### Immunohistochemistry and immunofluorescent staining

Formalin-fixed, paraffin-embedded tissues were cut into 4-μm sections and subjected to IHC and immunofluorescence (IF) staining as described before^43^. Antibodies used in the IHC and IF staining included: mouse mAb against human/mouse α-smooth muscle actin (α-SMA)-Cy3TM antibody (Cat no.#C6198, Sigma Aldrich (1:500 dilution)), p-AKT (Cat no.#4060S, Cell Signaling Technology (1 in 200 dilution)), p-PI3K (Cat no.#AF3242-50, Affinity (1 in 200 dilution)), rabbit mAb against human phospho-p65 (Ser536) (93H1) (Cat no.#3169S, Cell Signaling Technology (1:100 dilution)), rabbit mAb against human TGFα (Cat. no.#ab208156, Abcam (1 in 800 dilution)), PDGFRα (Cat no.#3174S, Cell Signaling Technology (1 in 100 dilution)), Ki67 (Cat no.#GB111499, Servicebio Technology (1:100 dilution)), p-EGFR (Cat no.#2236S, Cell Signaling Technology (1 in 100 dilution)), cleaved caspase-3 (Cat no.#9661S, Cell Signaling Technology (1 in 400 dilution)) and p-p53 (Cat no.#9284S, Cell Signaling Technology (1 in 100 dilution)) overnight at 4 °C. Antigen retrieval was performed under high pressure condition for 10 min in the presence of unmasking buffer (Tris-HCL, pH = 9.2). Finally, the immunodetection was done on the following day using DAB immunostaining kit (Dako) according to the manufacturer’s instruction. For the IF staining, alexa fluor–conjugated secondary antibodies (Invitrogen Molecular Probes) were used after the primary antibody incubation. DAPI was then used for counterstaining the nuclei and images were obtained by laser scanning confocal microscopy (MICA, Leica). The quantification of the staining was done as described previously^43^.

### Coverslip cell staining

sCAFs or rCAFs were disseminated in a concentration of 20,000 cells/well on glass slides in 24-well plates overnight and fixated with 4% paraformaldehyde for 10 min at room temperature. The permeabilization and blocking steps were performed by incubating the slides in blocking solution containing PBS (pH 7.4, 25 °C)/0.1% triton-X-100/3% BSA for 30 min, which were then incubated with mouse mAb against human/mouse α-smooth muscle actin (α-SMA)-Cy3TM antibody (Cat no.#C6198-2ML, Sigma Aldrich (1 in 500 dilution)), rabbit mAb against human PDGFRα (Cat no.#3174, Cell Signaling Technology (1 in 1000 dilution)), rabbit mAb against human CD13 (Cat no.#ab108310, Abcam (1 in 800 dilution)), rabbit mAb against human CD34 (Cat no.#ZA-0550-0.1, ZSGB-BIO (1 in 200 dilution)) at 4 °C for 12 h. Alexa Fluor–conjugated secondary antibodies and DAPI were used as described above.

### Cell viability assay

CCK8 (cell counting kit-8) was carried out according to the manufacturer’s protocol (Jiangsu KeyGENBioTECH Corp., Ltd). In brief, 2000–4000 cancer cells or sCAFs or rCAFs were treated with a range of different cisplatin or 5’FU concentrations for two or three days. For the co-culture experiments, 3 × 10^4^ FaDu or Tu686 cells were seeded in the lower chamber and 3 × 10^4^ sCAFs or rCAFs in the upper chamber of a 24-well transwell apparatus with 0.4 um pore size (Corning Incorporated, NY, USA) for 6 days, which were then harvested and plated onto 96-well plates overnight and subjected to DDP or 5’FU IC_50_ dose analysis. For the rescue experiments, FaDu or Tu686 cells were treated with a range of different cisplatin or 5’FU concentrations in the presence of CM from CAFs together with 100 μg/mL IgG control antibody or 100 μg/mL cetuximab for 48–72 h. For the heat inactivation experiment, the CM harvested from rCAFs was inactivated by heating at 56 °C for 2 h, which were subsequently cooled down to 37 °C before use.

### Cell counting experiment

FaDu and Tu686 cells were first transfected with a lentiviral vector encoding GFP (purchased from Genepharma) in the presence of 6 μg/mL polybrene at 37 °C, which were then selected with 2 μg/mL puromycin (Cat no.#ant-pr-1, InvivoGen) for 2 weeks. Similarity, sCAFs and rCAFs were stably transfected with an RFP lentiviral vector (purchased from Genepharma). Afterwards, GFP fluorescently labeled cancer cells were co-cultured with RFP fluorescently labeled sCAFs or rCAFs at 1:3 ratio for 48 h. The number of cancer cells or CAFs was counted under a fluorescent microscope.

### Colony formation assay

Cancer cells were placed at 500 cells/well in triplicate into 6-well plates and cultured in the presence of CM harvested from sCAFs or rCAFs together with or without 3 μM DDP in a humified incubator at 37 °C with 5% CO_2_ for 2 weeks, and then fixed with 4% formaldehyde for 30 min and stained with 0.1% crystal violet at room temperature for 1 h. Cell clusters with more than 50 cells were counted as a colony under a microscope.

### Transwell migration and invasion assays

For the fibroblast-based transwell migration assay, 5 × 10^4^ sCAFs or rCAFs were plated onto the upper chamber of each transwell insert with 8 μm pore size in a 24-well plate containing serum-free medium in the presence or absence of 8 μM DDP or 10 μM 5’FU. The lower chamber was filled with medium containing 20% FBS as a chemoattractant. After incubation for 48 h at 37 °C, non-migrated cells in the upper chamber were gently removed with cotton swabs, while the migrated cells on the lower chamber surface were fixed with 4% paraformaldehyde for 15 min and subsequently stained with 0.1% crystal violet at room temperature for another 15 min. For the transwell co-culture migration assay, 5 × 10^4^ cancer cells were seeded, with or without 1.67 × 10^4^ sCAFs or rCAFs, into the upper chamber of each transwell insert with 8 μm pore size in a 24-well plate containing serum-free medium in the presence or absence of DDP (FaDu:3 μM, Tu686:2 μM) or 5’FU (FaDu:15 μM, Tu686:7 μM). For the CM-based transwell migration experiment, 5 × 10^4^ cancer cells were seeded into the upper chamber of each transwell insert with 8 μm pore size in a 24-well plate containing with or without CM from sCAFs or rCAFs in the presence of DDP (FaDu:3 μM, Tu686:2 μM) or 5’FU (FaDu:15 μM, Tu686:7 μM). For the transwell invasion assay, a similar protocol to the migration assay was followed, except the upper transwell chamber was pre-coated with Matrigel. Following both assays, the crystal violet-stained cells that migrated or invaded were observed and photographed under a microscope. ImageJ software analysis was used to quantify the relative number of migrated or invaded cells.

### Transcriptomics analysis

sCAFs or rCAFs isolated from three different chemo-sensitive or -resistant HNSCC patients were harvested and lysed for total RNA extraction. The transcriptomics analysis of these samples was done using the service provided by Aksomics (China), while the illumine software BasseCaller was used to analyze and transform the sequence images, which were then demultiplexed to fastq files. The quality of the sequencing data was evaluated by using FastQC software, while the candidate genes were filtered and selected using an FDR-corrected *p*-value threshold of 0.05. The results were uploaded onto NCBI-GEO database (PRJNA905887).

### Proteomics analysis

The proteomics analysis was conducted using previously established methods^44^. Briefly, sCAFs or rCAFs were subjected to protein extraction using a cell lysis buffer that contained a protease and phosphatase inhibitor cocktail (Cat# PPC1010-5ML, Sigma Aldrich). Acetone was used at a 1:4 ratio to precipitate the protein, which was then dissolved in an 8 M urea solution (pH 8.5) at 37 °C for 1.5 h. The protein was reduced using 2 mM DTT for 1.5 h and alkylated with 12 mM IAA in darkness for 45 min. The samples were then digested overnight with Pierce™ Trypsin Protease, MS Grade (Cat# 90057, ThermoFisher Scientific). The reaction was terminated by adding trifluoroacetic acid to a final concentration of 0.4%, and the peptide solution was desalted with a reversed C18 spin column. The dried peptides were finally resuspended in 0.1% FA and subjected to LC-MS/MS analysis using the Thermo Scientific^TM^ Orbitrap Fusion^TM^, following the manufacturer’s instructions.

### Tissue culture

FaDu and Tu686 cell lines were purchased from American Type Culture Collection (ATCC, Manassas, VA, USA) and were cultured in DMEM or RPMI1640 supplemented with 10% fetal bovine serum and 1% penicillin and streptomycin and grown in a 5% CO_2_ incubator at 37 °C. For co-culture experiments, 1 × 10^5^ stable GFP overexpressing Fadu or Tu686 cells and 3 × 10^5^ stable RFP expressing sCAFs or rCAFs were seeded onto a 12-well plate overnight, which were then treated with either placebo, cisplatin or 5’FU for 48 h. Afterwards, the fluorescently labeled cells were imaged and counted using an immunofluorescent microscope. For AKT or p65 inhibitor experiment, rCAFs were treated with either placebo, AKT (10 μM Perifosine) or p65 (20 μM Maslinic acid) inhibitor together with 8 μM cisplatin or 15 μM 5’FU for 12 h. For siRNA transfection experiment, rCAFs were transiently transfected with 50 nM TGFα targeting siRNA-1/-2 or non-silencing control siRNA (GenePharma) using lipofectamine 3000® transfection reagent (Invitrogen). The list of all siRNA used in this study, including names and sequences, was given in Supplementary Table 1.

### Quantitative real-time PCR

Total RNA was extracted from cancer cells/CAFs using TRIzol reagent (Invitrogen, AM9738). By using 5 X All-in-One RT MasterMix (Cat no.#G492, Abm), 2 μg of total RNA was reversely transcribed into cDNA and followed by quantitative real-time PCR (q-PCR) using HieffTM qPCR SYBR® Green MasterMix (Cat no.#11201ES08, NO ROX, YEASEN). All PCR reactions were run in triplicate and performed on a Roche Light Cycler 480 instrument II (Roche Diagnostic). The primers used for qPCR are provided in Supplementary Table 1.

### Western blotting

Cancer cells, sCAFs or rCAFs were first harvested and lysed with NP40 lysis buffer (Invitrogen), while the protein concentration of their lysates was measured by using the Bio-Rad DC protein assay kit (Bio-Rad Laboratories). In total, 20–30 μg of protein from each sample was loaded onto 8–12% polyacrylamide gels, while the protein was later transferred to a nitrocellulose membrane and incubated with 5% milk in PBS 0.1% Tween-20 (PBST) Afterwards, the membrane was incubated with indicated primary antibodies in 2% milk with PBST overnight at 4 °C. The blots were then washed three times with PBST and incubated with the corresponding HRP-conjugated second antibody diluted 1:2000 in 2% milks with PBST for 1 h at room temperature. The chemiluminescence of the blots were detected by using Mini Chemiluminescent Imaging and Analysis System (Sagecreation). Primary antibodies used in this study included: anti-TGFα (Cat no.#ab208156, Abcam (1 in 1000 dilution)), anti-EGFR (Cat no.#2232s, Cell Signaling Technology (1 in 1000 dilution)), anti-p-EGFR (Cat no.#2236S, Cell Signaling Technology (1 in 1000 dilution)), anti-p-AKT (Cat no.#4060S, Cell Signaling Technology (1 in 1000 dilution)), anti-total AKT (Cat no.#4691S, Cell Signaling Technology (1 in 1000 dilution)), anti-total PI3K (Cat no.#20584-1-AP, Proteintech (1 in 1000 dilution)), anti-p-PI3K (Cat no.#AF3242–50, Affinity (1 in 1000 dilution)), anti-p-Src (Cat no.#2101S, Cell Signaling Technology (1 in 1000 dilution), anit-total Src (Cat no.#2109S, Cell Signaling Technology (1 in 1000 dilution), anti-cleaved caspase-3 (Cat no.#9661S, Cell Signaling Technology (1 in 1000 dilution)), anti-p-p53(Cat no.#9284S, Cell Signaling Technology (1 in 1000 dilution)), total p53(Cat no.#sc-126, Santa Cruz (1 in 1000 dilution), p-p65(Cat no.#3033S, Cell Signaling Technology (1 in 1000 dilution)), total p65(Cat no.#8242S, Cell Signaling Technology (1 in 1000 dilution)). β-actin (Cat no.#sc-47778, Santa Cruz (1 in 5000 dilution) was used as a loading control. All blots originate from the same experiment and have undergone parallel processing. All uncropped blots were included in Supplementary Fig. 7.

### Enzyme-linked immunosorbent assay

The patients’ sera were used to perform ELISA to determine TGFα level following the manufacturer’s recommendations (Cat no.#JM-03246H2, JINGMEI). The TGFα level in each sample was calculated according to the standard curve after subtracting the negative control signal when measuring the level of TGFα in each patient’s serum sample.

### Cytokine array

Proteome Profiler Human XL Cytokine array was carried out according to the manufacturer’s instruction (Cat no.#ARY022B, R&D systems). Briefly, 250 μg of whole cell lysate from sCAFs or rCAFs was incubated per membrane.

### Apoptosis assay

sCAFs or rCAFs were treated with either 8 μM cisplatin, 15 μM 5’FU or 0.1% DMSO (as a control) in the presence or absence of placebo, 10 μM AKT (Perifosine) or 20 μM p65 (Maslinic acid) inhibitors for 48 h. After the harvest, cells were subsequently treated with Annexin V-FITC/PI Apoptosis Kit (Cat no. #6409914, Biolegend) and finally analyzed by using a FCM (BD Accuri™ C6). The FACS gating strategies were given in Supplementary Fig. 8b.

### Chromatin Immunoprecipitation experiment

ChIP assay was performed by using ChIP assay kits (Cat no.#17–10086, Merck Millipore) according to the manufacturer’s instructions. In short, 1 × 10^6^ sCAFs and rCAFs were first plated onto 10 cm dishes and left untouched overnight respectively. Sub-confluent cells were then fixed with 4% formaldehyde. After 10 min, the cross-linking reaction was instantly stopped by adding 90 mM glycine and washed with cold PBS three times, which were then harvested in PBS supplemented with protease and phosphatase inhibitors available from the kit. Chromatin was then subjected to sonication to generate DNA fragments. Antibodies used for ChIP in this study included: anti-p65 (Cat no.# 8242S, Cell Signaling Technology) and IgG control (Cat no.#12–371, Merck Millipore). The RT-PCR primer sequences of 1st/2nd putative p65 binding site were given in Table S1.

### Patient tumor derived organoids

HNSCC patient tumor derived organoids were generated as previously described with some modifications^45^. Briefly, tumors were first cut and minces into small pieces, which were then incubated in a digestion medium (including 1 mg/mL Collagenase XI, 10 μg/mL DNAase I, 10.5 μmol/L Y-27632 in human complete medium) at 37 °C for 30 min, the supernatant was collected and filtered through 70 μm filters and centrifugated at 800 rpm for 5 min. The pellet was then resuspended in Matrigel matrix (Corning) and seeded onto a 24-well plate for 15 min inside a CO_2_ incubator at 37 °C. Finally, each well of the 24-well plate was incubated with 1 mL of tumor organoid culturing medium, which included advanced DMEM/F12 medium containing HEPES 10 mmol/L, 1X Glutmax, A83–01 500 nmol/L, hEGF 50 ng/mL, mNoggin 100 ng/mL, hFGF10 100 ng/mL, hGastrin I 0.01 μmol/L, nicotinamide 10 mmol/L, N-acetylcysteine 1.25 mmol/L, PGE2 1 μmol/L, B27 supplement, R-spondin1 conditioned media, and Afamin/Wnt3A conditioned media. For co-culture experiment, sCAFs or rCAFs were first seeded onto a 24-well plate overnight, while organoids mixed with Matrigel matrix (Corning) were then placed on top of these cells in the presence or absence of DDP -/+ cetuximab.

### Animal model

4- to 6-week-old nude mice were subcutaneously injected with 1 × 10^6^ FaDu cells either alone or co-injected with 1 × 10^6^ FaDu cells and 3 × 10^6^ sCAFs or rCAFs in a 1 to 3 ratio. Animal surgery was performed following the induction of anesthesia with sodium pentobarbital (40 mg/kg) via intraperitoneal (IP) injection. Once the mean tumor volume had reached around 100 mm^3^, the tumor-bearing mice were randomly assigned to different groups. These groups were then intraperitoneally injected with either a placebo, 6 mg/kg DDP, IgG control (Cat no.#ab109489, Abcam), 15 mg/kg cetuximab (Hansoh Pharma), DDP + IgG control, or DDP + cetuximab, with injections administered every 6 days for up to four times in 18 days. Tumor size was monitored every 4–6 days using a caliper, and the tumor volume (V) was calculated using the formula: V = ½ (Length × Width^2^). Tumor growth was assessed by measuring tumor size with a calliper on a weekly basis. At the experimental endpoint, all nude mice were euthanized via CO_2_ asphyxiation. Afterward, their tumors were meticulously dissected, weighed, and then processed for immunohistochemical and immunofluorescent analyses, involving fixation, paraffin embedding, and subsequent sectioning. All animal procedures were approved by the Institutional Animal Care and Use Committee (IACUC) of Sun Yat-sen University, and they adhered to the ARRIVE guidelines.

### General statistical analysis

Based on the data type, unpaired student’s *t* test, one-way ANOVA, two-way ANOVA, log-rank test was used in this study. The n number of each experiment and the statistical test used was stated in the figure legend. All the in vitro experiments were repeated three times independently unless otherwise stated in the figure legend, and the results were normalized to the control for each experiment. The *n* numbers of animal experiments were given in the figure legend. The *n* number of patient cohort was also given in the figure legend. The statistical analysis was done by using Prism GraphPad software. The data are presented as means ± standard error, unless otherwise indicated in the figure legend. Values of *P* < 0.05 were considered statistically significant in this study.

### Reporting summary

Further information on research design is available in the Nature Research Reporting Summary linked to this article.

### Supplementary information

Supplementary information

REPORTING SUMMARY

## Data Availability

The RNA-seq data generated in this study were deposited in the GEO under the accession number PRJNA905887. The proteomics data were deposited in the iProx database under the accession number PXD038343 (Web link: https://www.iprox.cn/page/PSV023.html;?url=1669253545524hzBg). The data that supports the finding of this study are included in the article or supplementary information. Additional data supporting the findings of this study are available from the corresponding author upon request.
