# Supplementary material for: Chemoresistant fibroblasts dictate neoadjuvant chemotherapeutic response of head and neck cancer via TGFα-EGFR paracrine signaling
Source: NPJ Precis Oncol. 2023 Oct 11;7:102. doi: 10.1038/s41698-023-00460-2 (PMC10567732; doi:10.1038/s41698-023-00460-2)
Supplement: Supplementary file 1 — Supplementary information [file 41698_2023_460_MOESM1_ESM.pdf]

Supplementary information

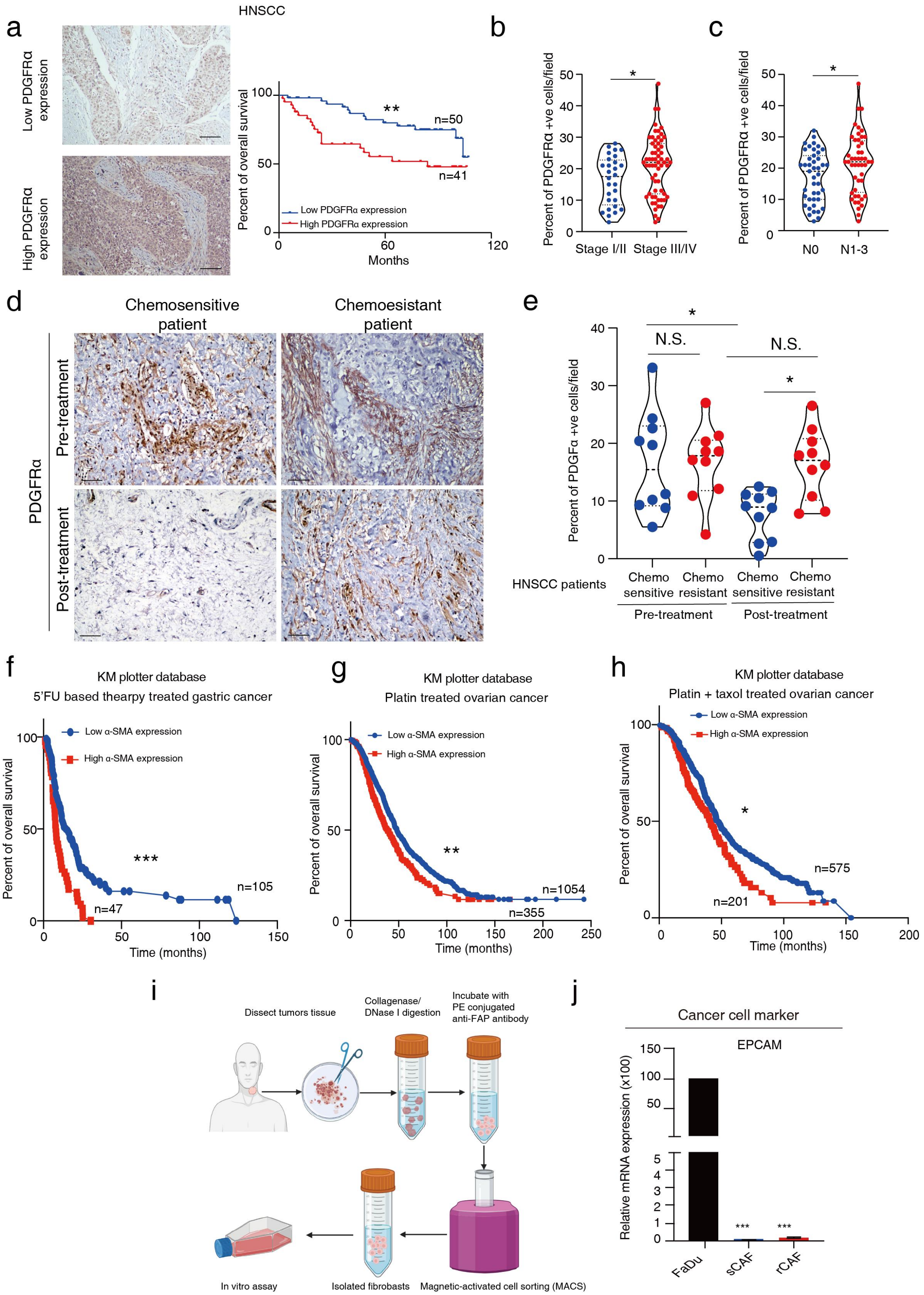

Supplementary Fig. 1: High CAF population predicts poor outcomes and reduced therapeutic response in cancer patients. (**a**) IHC staining of PDGFRα in tumor sections derived from HNSCC patients. Kaplan-Meier survival study of HNSCC patients with high or low PDGFRα expression (n=91 patients, our cohort). (**b, c**) Violin plots showing the correlation between PDGFRα staining intensity and tumor size or cancer progression in HNSCC patients (n= 91 patients, our cohort). (**d, e**) IHC staining of PDGFRα in tumor sections derived from chemo-sensitive and -resistant HNSCC patients. Violin plots show the intensity of PDGFRα staining in each group (n=20 patients, our cohort). (**f-h**) Kaplan-Meier plotter database analysis showing the relationship between α-SMA expression and patient survival in chemotherapy treated gastric and ovarian cancer patients (n=152 5'FU based therapy treated gastric cancer patients; n=1409 platin treated ovarian cancer patients; n=776 platin + taxol treated ovarian cancer patients). (**I**) Schematic diagram representing the isolation and purification method of CAFs from HNSCC patient derived pre-treatment tumor biopsies. (**j**) RT-PCR analysis of the cancer cell marker EPCAM in sCAFs and rCAFs as compared to FaDu cancer cells. *P<0.05, **P<0.01, ***P<0.001, N.S. non-significant. (**a, f-h**) Log-rank (Mantel-Cox) test. (**b, c**) Student's t test. (**e, j**) One-way ANOVA. Scale bars in (**a, d**) represent 100 μm.

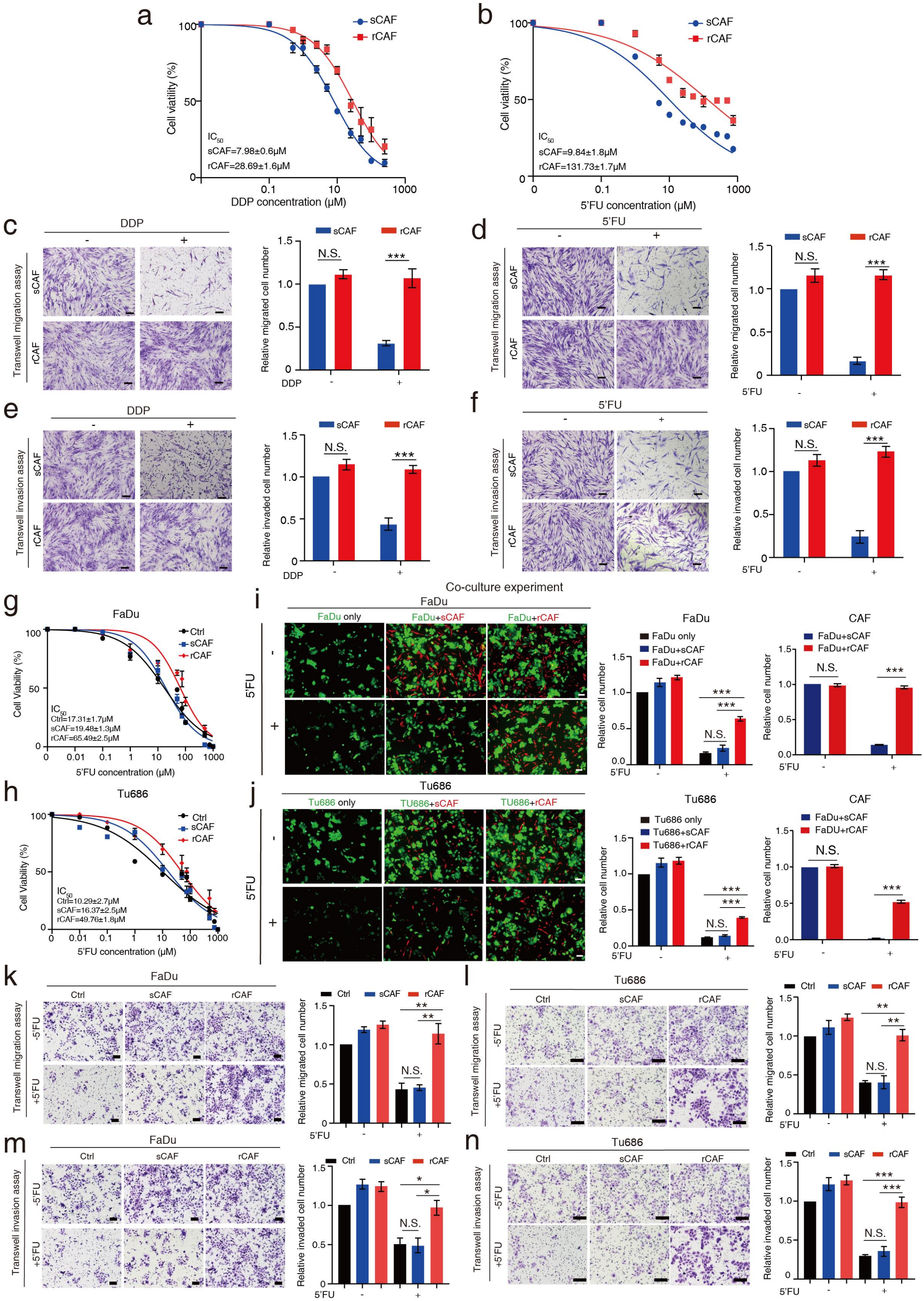

a
sCAF
rCAF
Cell viatility (%)
DDP concentration (μM)
IC50
sCAF=7.98±0.6μM
rCAF=28.69±1.6μM
b
5'FU concentration (μM)
sCAF=9.84±1.8μM
rCAF=131.73±1.7μM
c
DDP
Transwell migration assay
Relative migrated cell number
N.S.
***
d
5'FU
e
Transwell invasion assay
Relative invaded cell number
f
Co-culture experiment
g
FaDu
Ctrl
Cell Viability (%)
5'FU concentration (μM)
Ctrl=17.31±1.7μM
sCAF=19.48±1.3μM
rCAF=65.49±2.5μM
h
Tu686
Ctrl=10.29±2.7μM
sCAF=16.37±2.5μM
rCAF=49.76±1.8μM
i
FaDu only
FaDu+sCAF
FaDu+rCAF
CAF
Relative cell number
j
Tu686 only
TU686+sCAF
TU686+rCAF
Tu686+sCAF
Tu686+rCAF
FaDu+sCAF
FaDU+rCAF
k
-5'FU
+5'FU
**
l
m
*
n

Supplementary Fig. 2: rCAFs are more resistant to chemotherapy treatment as compared to sCAFs. (**a, b**) DDP or 5'FU IC_{50} experiments of sCAFs and rCAFs. Data are shown as means ± S.D. (**c-f**) Transwell migration or invasion assays of sCAFs and rCAFs in the presence or absence of DDP or 5'FU. (**g, h**) 5'FU IC_{50} experiments of FaDu and Tu686 cells after either co-culture -/+ sCAFs or rCAFs. (**i, j**) Representative immunofluorescent images are given as indicated. Bar charts show the number of GFP overexpressing tumor cells and RFP expressing sCAFs or rCAFs after treated with or without 5'FU. (**k-n**) Transwell migration or invasion assays of FaDu and Tu686 cells after co-culture -/+ sCAFs or rCAFs in the presence or absence of 5'FU. Data are shown as means ± S.E.M. **P*<0.05, ***P*<0.01, ****P*<0.001, N.S. non-significant. (**c-f, I, j, k-n**) One-way ANOVA. Scale bars in (**c-f, i-n**) represent 100 μm.

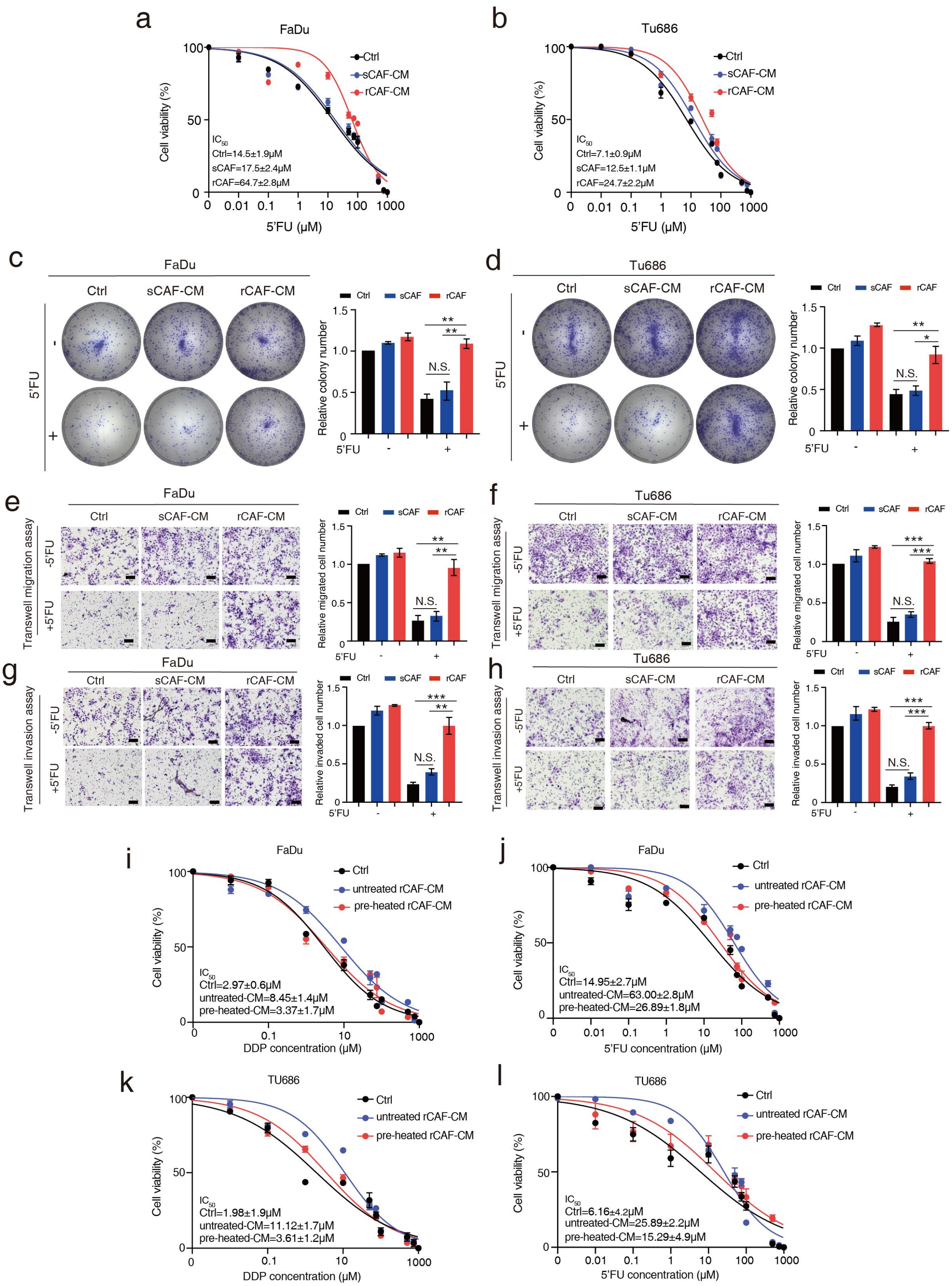
a
FaDu
Ctrl
sCAF-CM
rCAF-CM
Cell viability (%)
IC50
Ctrl=14.5±1.9μM
sCAF=17.5±2.4μM
rCAF=64.7±2.8μM
5'FU (μM)
b
Tu686
Ctrl
sCAF-CM
rCAF-CM
Cell viability (%)
IC50
Ctrl=7.1±0.9μM
sCAF=12.5±1.1μM
rCAF=24.7±2.2μM
5'FU (μM)
c
FaDu
Ctrl
sCAF-CM
rCAF-CM
5'FU
Relative colony number
N.S.
d
Tu686
Ctrl
sCAF-CM
rCAF-CM
5'FU
Relative colony number
N.S.
e
FaDu
Transwell migration assay
-5'FU
+5'FU
Relative migrated cell number
N.S.
f
Tu686
Transwell migration assay
Relative migrated cell number
g
FaDu
Transwell invasion assay
Relative invaded cell number
h
Tu686
Transwell invasion assay
Relative invaded cell number
i
FaDu
Ctrl
untreated rCAF-CM
pre-heated rCAF-CM
IC50
Ctrl=2.97±0.6μM
untreated-CM=8.45±1.4μM
pre-heated-CM=3.37±1.7μM
DDP concentration (μM)
j
FaDu
IC50
Ctrl=14.95±2.7μM
untreated-CM=63.00±2.8μM
pre-heated-CM=26.89±1.8μM
5'FU concentration (μM)
k
TU686
IC50
Ctrl=1.98±1.9μM
untreated-CM=11.12±1.7μM
pre-heated-CM=3.61±1.2μM
DDP concentration (μM)
l
TU686
IC50
Ctrl=6.16±4.2μM
untreated-CM=25.89±2.2μM
pre-heated-CM=15.29±4.9μM
5'FU concentration (μM)

Supplementary Fig. 3: rCAF enhances chemoresistance and tumorigenesis via paracrine factors. (**a, b**) 5'FU IC_{50} experiments of FaDu and Tu686 cells after exposed with CM from sCAFs and rCAFs respectively. (**c, d**) Colony formation assay of FaDu and Tu686 cells after exposed with CM from sCAFs or rCAFs in the presence or absence of 5'FU. (**e-h**) Transwell migration or invasion assays of FaDu and Tu686 cells after exposed with CM from sCAFs or rCAFs in the presence or absence of 5'FU (n=3 independent experiments). Data are shown as means ± S.E.M. (**i-l**) DDP or 5'FU IC_{50} experiments of FaDu and Tu686 cells after treated with or without heat inactivated (pre-heated) or untreated CM from rCAFs in the presence or absence of 5'FU. Ctrl represents untreated cancer cells. ***$P<0.001$. (**c-h**) One-way ANOVA. Scale bars in (**e-h**) represent 100 μm.

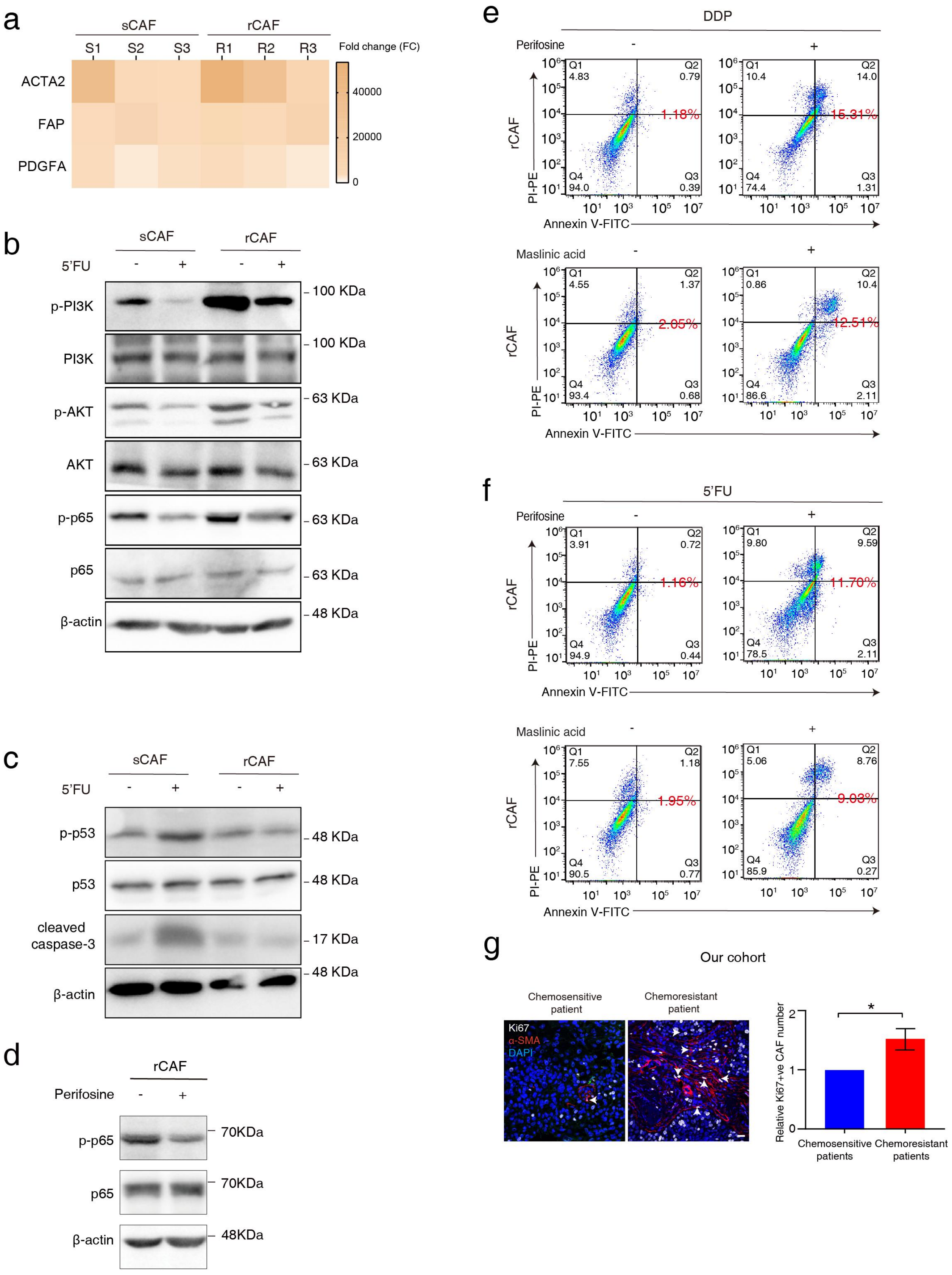

a
sCAF
rCAF
S1
S2
S3
R1
R2
R3
Fold change (FC)
ACTA2
FAP
PDGFA
40000
20000
0
b
sCAF
rCAF
5'FU
- + - +
p-PI3K
100 KDa
PI3K
100 KDa
p-AKT
63 KDa
AKT
63 KDa
p-p65
63 KDa
p65
63 KDa
β-actin
48 KDa
c
sCAF
rCAF
5'FU
- + - +
p-p53
48 KDa
p53
48 KDa
cleaved caspase-3
17 KDa
β-actin
48 KDa
d
rCAF
Perifosine
- +
p-p65
70KDa
p65
70KDa
β-actin
48KDa
e
DDP
Perifosine
- +
rCAF
PI-PE
Annexin V-FITC
Q1 4.83
Q2 0.79
Q4 94.0
Q3 0.39
1.18%
Q1 10.4
Q2 14.0
Q4 74.4
Q3 1.31
15.31%
Maslinic acid
Q1 4.55
Q2 1.37
Q4 93.4
Q3 0.68
2.05%
Q1 0.86
Q2 10.4
Q4 86.6
Q3 2.11
12.51%
f
5'FU
Perifosine
Q1 3.91
Q2 0.72
Q4 94.9
Q3 0.44
1.16%
Q1 9.80
Q2 9.59
Q4 78.5
Q3 2.11
11.70%
Maslinic acid
Q1 7.55
Q2 1.18
Q4 90.5
Q3 0.77
1.95%
Q1 5.06
Q2 8.76
Q4 85.9
Q3 0.27
9.03%
g
Our cohort
Chemosensitive patient
Chemoresistant patient
Ki67
α-SMA
DAPI
Relative Ki67+ve CAF number
*
Chemosensitive patients
Chemoresistant patients

Supplementary Fig. 4: Treatment with AKT or p65 inhibitor rescues the chemosensitivity in rCAFs. (**a**) Heatmap analysis of CAF related marker gene expression between three different patients derived rCAFs and sCAFs used in figure 4 (n=3 patients for each group). (**b, c**) Western blot analysis of the indicated protein expression in sCAFs and rCAFs after treated with or without 5'FU. (**d**) Western blot analysis of the indicated protein expression in rCAFs after treated with or without 10 μM AKT inhibitor. (**e, f**) Annexin-PI staining of sCAFs and rCAFs after treated with 5'FU or DDP -/+ AKT (Perifosine) or p65 (Maslinic acid) inhibitors (n= 3 independent experiments). (**g**) Co-immunofluorescent analysis of Ki67 and α-SMA expression in tumor sections derived from chemo-sensitive and -resistant patients (n=20 patients, our cohort). Arrow indicates Ki67 and α -SMA double positive cells. Bar chart shows the relative Ki67 positive CAF number in each group (n= 20 patients, our cohort). Data are shown as means ± S.E.M. Scale bar in (**f**) represents 100 μm.

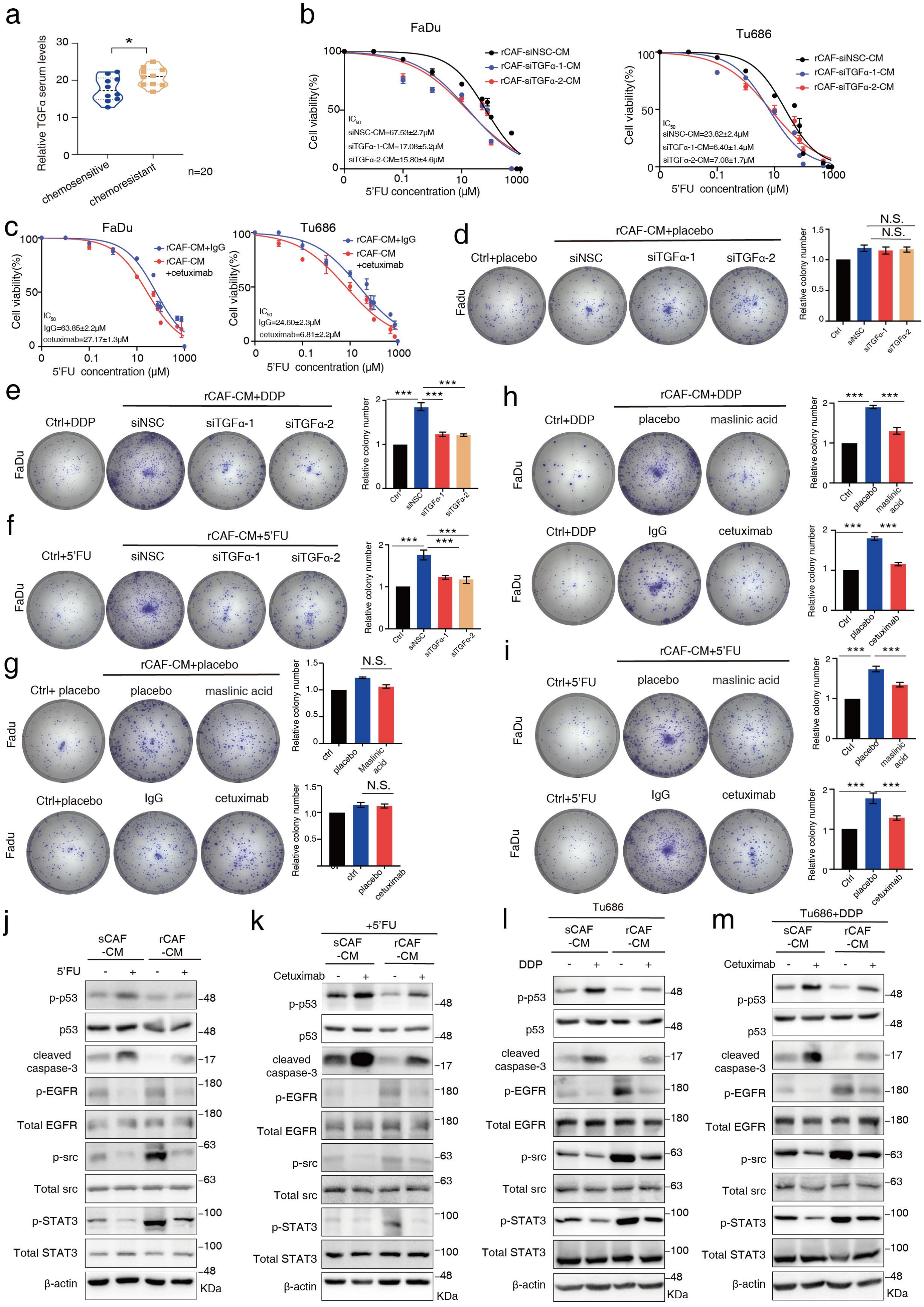
a
Relative TGFα serum levels
chemosensitive
chemoresistant
n=20
b
FaDu
rCAF-siNSC-CM
rCAF-siTGFα-1-CM
rCAF-siTGFα-2-CM
IC50
siNSC-CM=67.53±2.7μM
siTGFα-1-CM=17.08±5.2μM
siTGFα-2-CM=15.80±4.6μM
Cell viability(%)
5'FU concentration (μM)
Tu686
siNSC-CM=23.82±2.4μM
siTGFα-1-CM=6.40±1.4μM
siTGFα-2-CM=7.08±1.7μM
c
rCAF-CM+IgG
rCAF-CM +cetuximab
IgG=63.85±2.2μM
cetuximab=27.17±1.3μM
IgG=24.60±2.3μM
cetuximab=6.81±2.2μM
d
rCAF-CM+placebo
Ctrl+placebo
siNSC
siTGFα-1
siTGFα-2
Relative colony number
N.S.
e
rCAF-CM+DDP
Ctrl+DDP
f
rCAF-CM+5'FU
Ctrl+5'FU
g
Ctrl+ placebo
placebo
maslinic acid
IgG
cetuximab
h
i
j
sCAF -CM
rCAF -CM
5'FU
p-p53
p53
cleaved caspase-3
p-EGFR
Total EGFR
p-src
Total src
p-STAT3
Total STAT3
β-actin
KDa
k
+5'FU
Cetuximab
l
Tu686
DDP
m
Tu686+DDP

Supplementary Fig. 5: Depletion of TGFα by siRNAs or cetuximab in rCAF rescues its paracrine effect on cancer cell chemosensitivity. (**a**) ELISA assay of the serum level of TGFα in chemo-sensitive and -resistant patients before receiving the NACT treatment (n=10 patients per group). (**b**) 5'FU or DDP IC_{50} experiments of FaDu or Tu686 cells exposed with/without CM from siTGFα-1/-2 or siNSC transfected rCAFs (n= 3 experimental repeats). (**c**) 5'FU or DDP IC_{50} experiments of FaDu or Tu686 cells exposed with or without CM from rCAFs in the presence of IgG control or cetuximab. (**d-f**) Colony formation assays of FaDu cells after treated with CM from siTGFα-1/-2 or siNSC transfected rCAFs in the presence or absence of 5'FU or DDP. (**g-i**) Colony formation assays of FaDu cells after exposed with either CM from placebo or maslinic acid pre-treated rCAFs, or CM from rCAFs supplemented with IgG control antibody or cetuximab. Representative images of crystal violet-stained colonies in each group are given (n=3 independent experiments). (**j**) Western blot analysis of p-EGFR, total EGFR, p-Src, total Src, p-STAT3, and total STAT3 expression in FaDu cells after treated with CM from sCAFs or rCAFs in the presence or absence of 5'FU. (**k**) Western blot analysis of the indicated protein expression in FaDu cells after treated with 5'FU in the presence of CM from sCAFs or rCAFs together with or without cetuximab. (**l**) Western blot analysis of the indicated protein expression in Tu686 cells after treated with CM from sCAFs or rCAFs in the presence or absence of DDP. (**m**) Western analysis of the indicated protein expression in Tu686 cell after treated with DDP in the presence of CM from sCAFs or rCAFs, with or without cetuximab (n= 3 independent experiments). Data are shown as means ± S.E.M. ***P<0.001. N.S. non-significant difference. (**a**) Student's test. (**d-i**) One-way ANOVA.

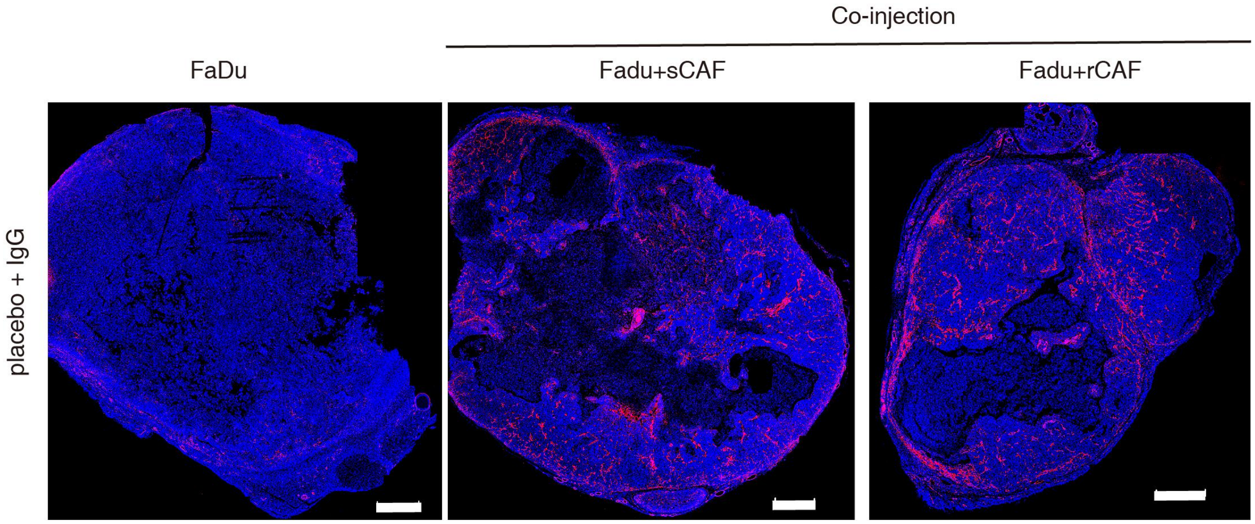

Supplementary Fig. 6: Increased CAF population was observed in FaDu tumors co-injected with sCAFs or rCAFs as compared to FaDu alone group. Representative images of immunoflouresecnt staining of α-SMA in tumor sections dervied from each group are shown. Scale bars represent 1 mm.

Figure 3G

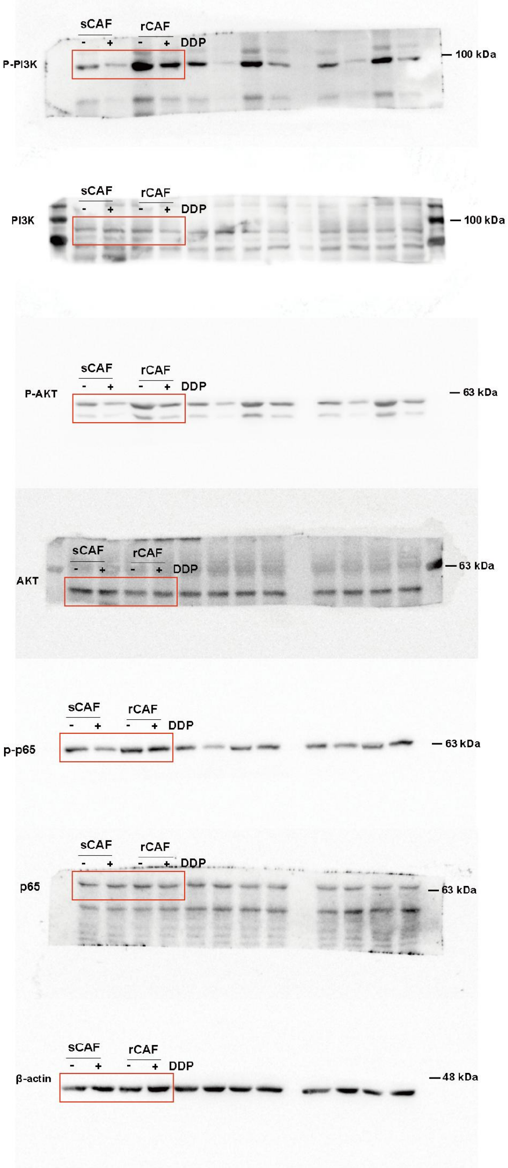

Figure 3H

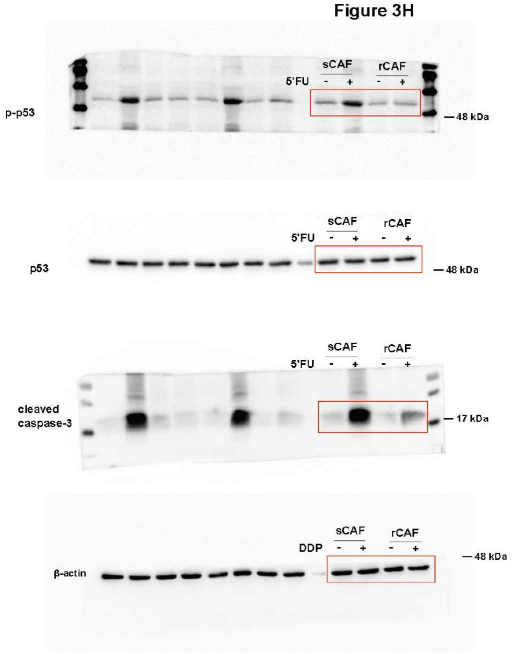

Figure 4L Figure 4M

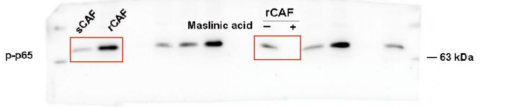

Figure 4L Figure 4M

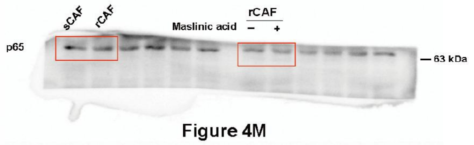

Figure 4M

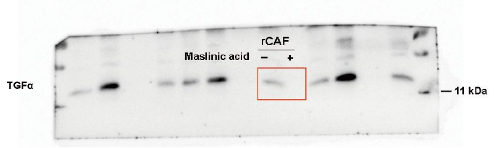

Figure 4L Figure 4M

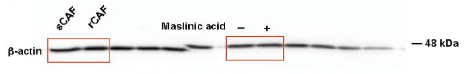

Figure 4N

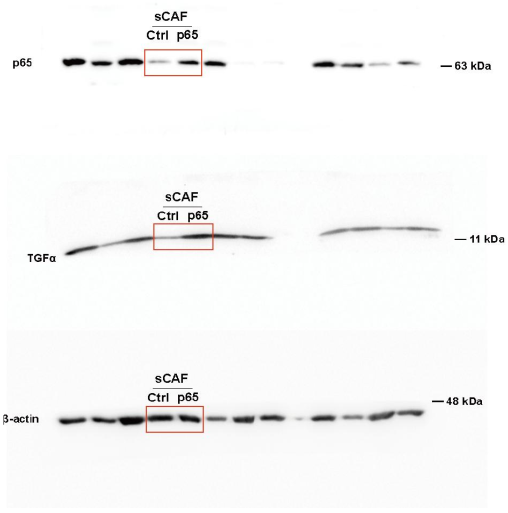

Figure 4C

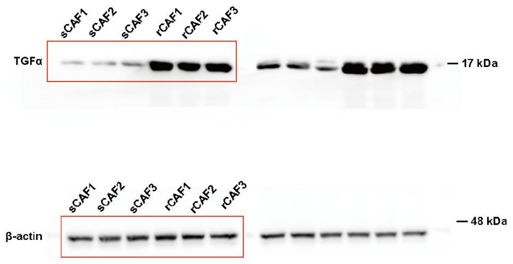

Figure 5G Supplementary figure 5J

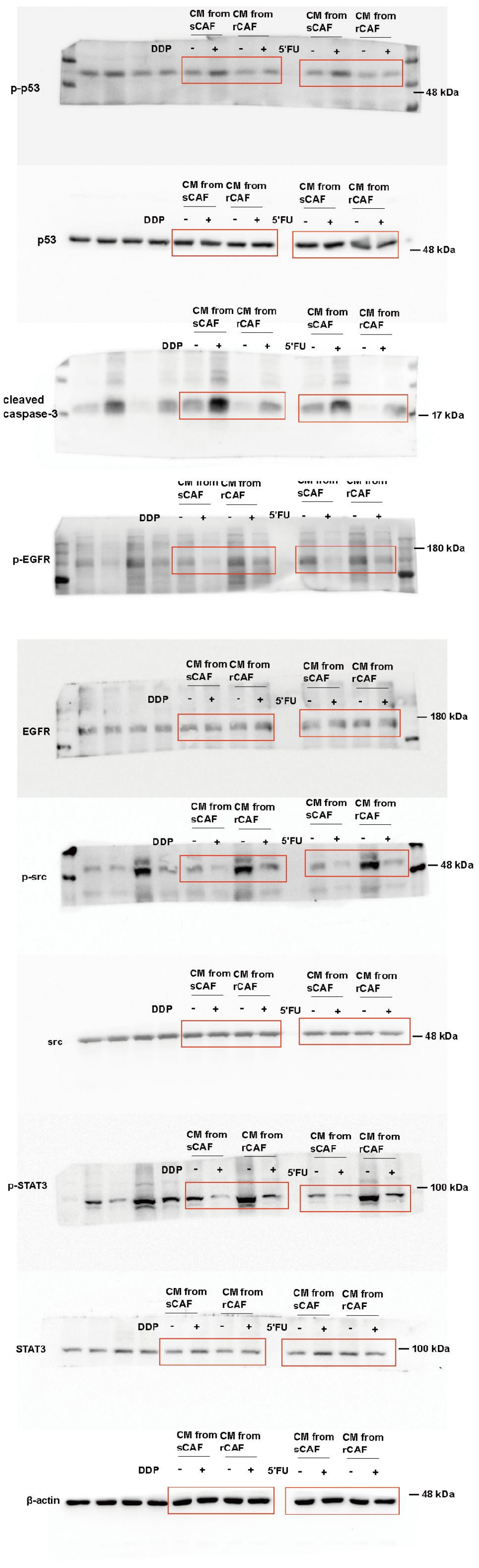

Figure 5H Supplementary figure 5K

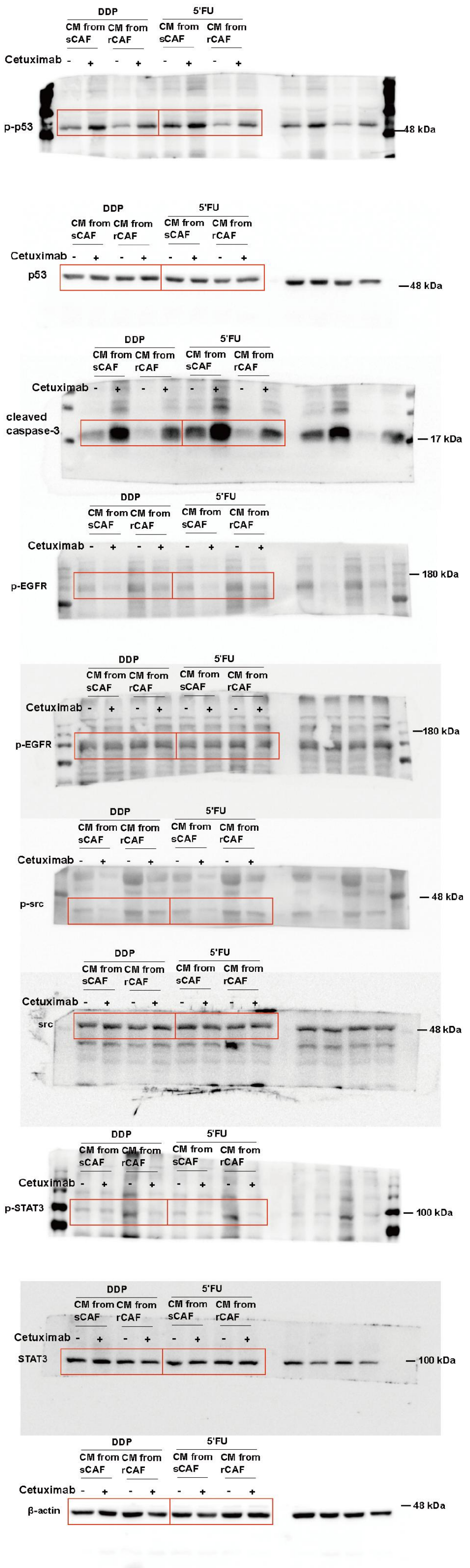

Figure 5A

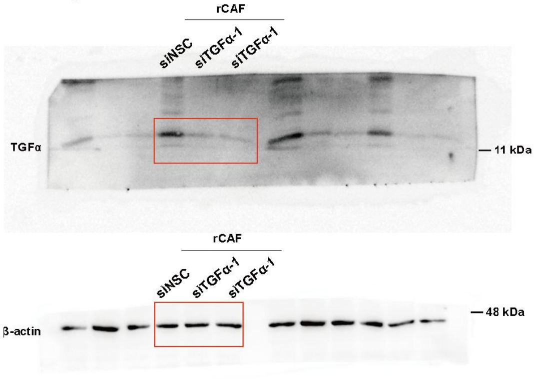

Supplementary figure 4B

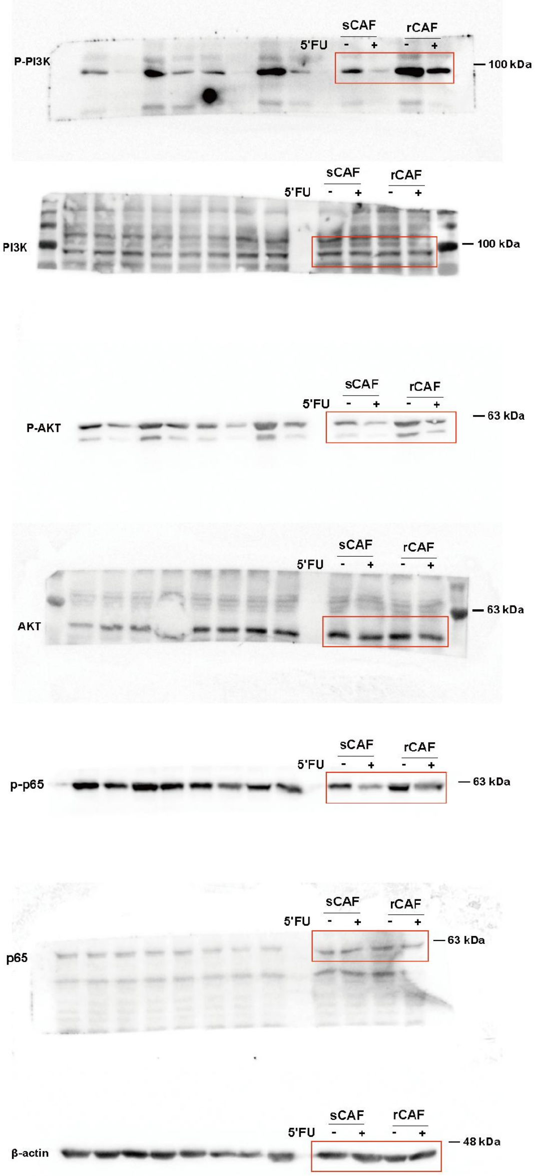

Supplementary figure 4C

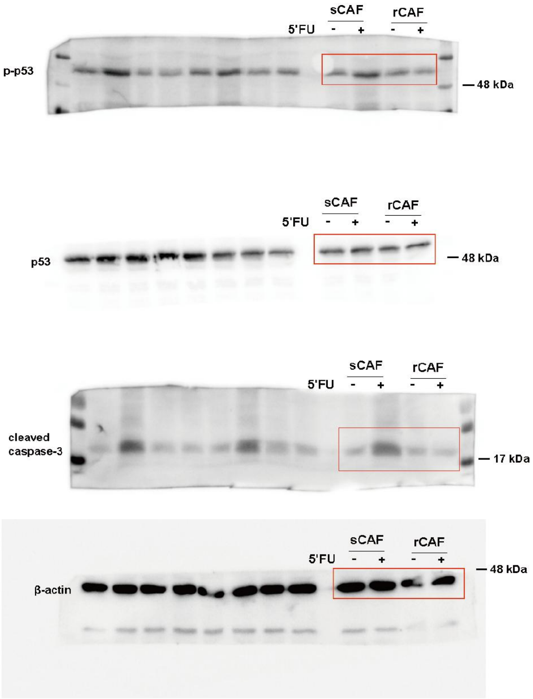

Supplementary figure 4D

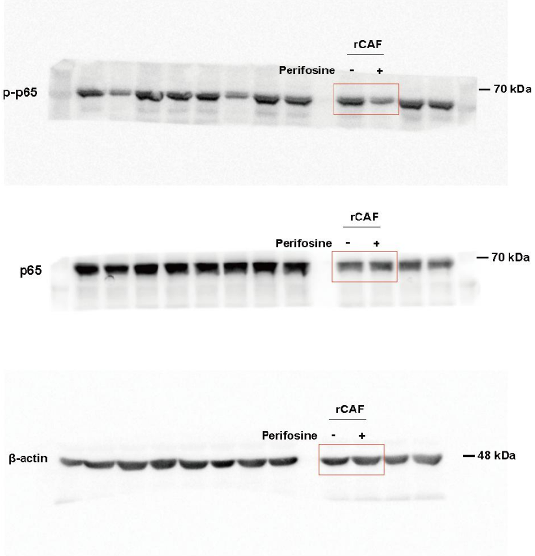

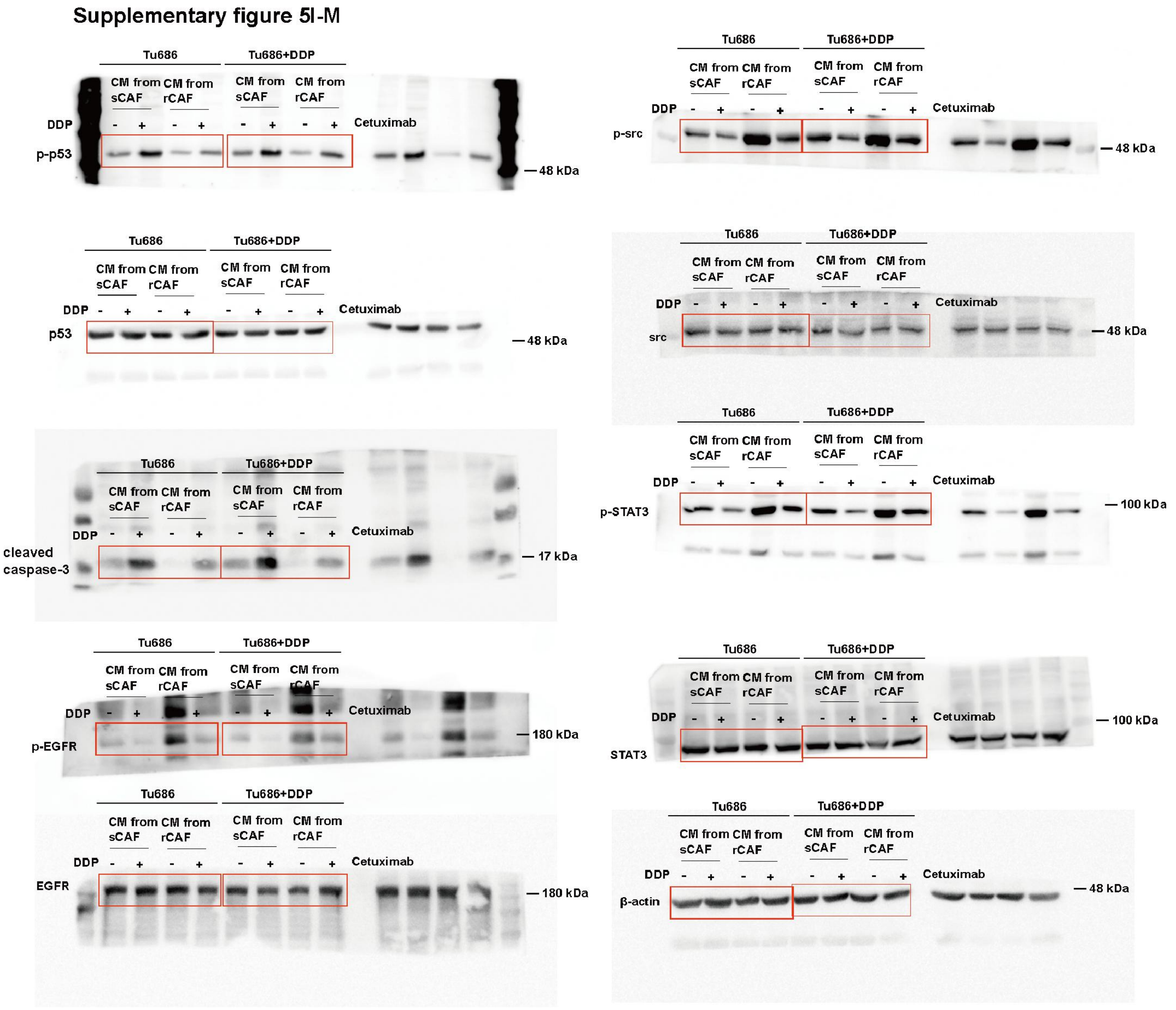

Supplementary Fig. 7: Uncropped blots for main figures. Blots shown in the main article and Supplementary Information are depicted by boxed regions in each of associated uncropped.

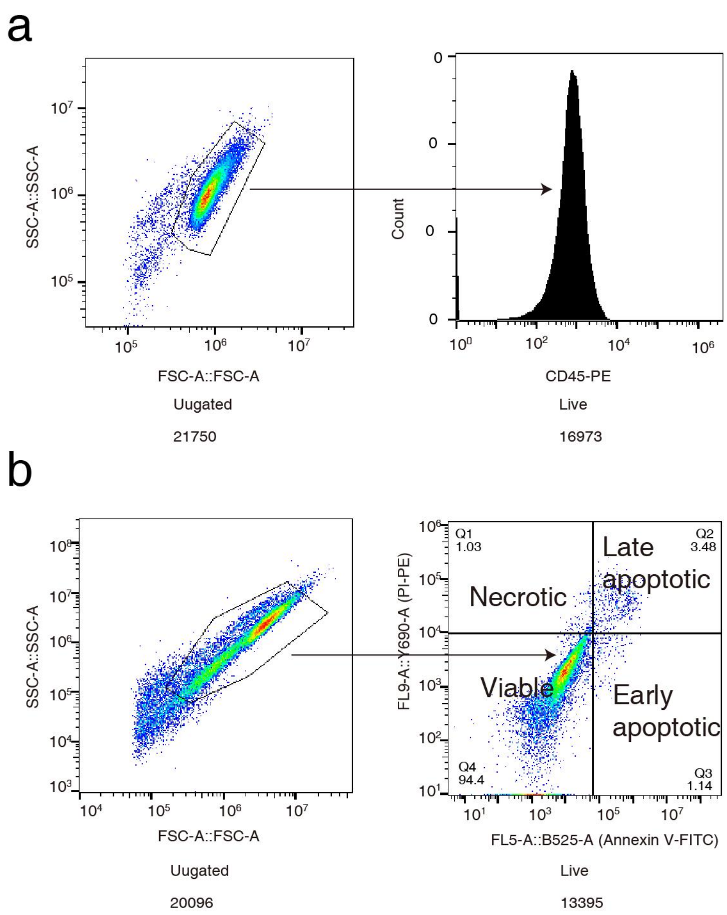

Supplementary Fig. 8: Flow cytometry gating strategies. (**A**) Gating strategy for the selection of antibody-stained sCAFs or rCAFs in Fig. 1g. (**B**) Gating for sorting propidium iodide (PI)/annexin V-stained sCAFs or rCAFs as presented in Fig. 3i and Supplementary Fig. 4e-f. The different quadrants represent distinct apoptotic statuses, categorized as follows: Q1 (necrotic), Q2 (late apoptotic), Q3 (early apoptotic), and Q4 (viable, non-apoptotic).

Supplementary Table 1. The primers used in RT-qPCR and siRNA.

Gene	Forward primers (5'-3')	Reverse primers (5'-3')
ICAM-1	TTGGGCATAGAGACCCCGTT	GCACATTGCTCAGTTCATACACC
THBS1	AGACTCCGCATCGCAAAGG	TCACCACGTTGTTGTCAAGGG
TGFα	AGGTCCGAAAACACTGTGAGT	AGCAAGCGGTTCTTCCCTTC
μPAR	GAGCTATCGGACTGGCTTGAA	CGGCTTCGGGAATAGGTGAC
VDBP	CCCAGTGGCACGTTTGAAC	CTGGTGTCATAGCAGTCAGGG
EGF	TGTCCACGCAATGTGTCTGAA	CATTATCGGGTGAGGAACAACC
EPCAM	AATCGTCAATGCCAGTGTACTT	TCTCATCGCAGTCAGGATCATAA
p65-proximal site	TCCCAGGTTCAAGTGATTCTCG	GCTGAGGTGGGTGGATTGC
hs-TGFA-si-1	GGCUGUCCUUAUCAUCACATT	UGUGAUGAUAAGGACAGCCTT
hs-TGFA-si-2	GUUCGCUCUGGGUAUUGUGUUTT	AACACAAUACCCAGAGCGAACTT
NC	UUCUCCGAACGUGUCACGUdTdT	ACGUGACACGUUCGGAGAAdTdT
