# Supplementary material for: Chemoresistant fibroblasts dictate neoadjuvant chemotherapeutic response of head and neck cancer via TGFα-EGFR paracrine signaling
Source: NPJ Precis Oncol. 2023 Oct 11;7:102. doi: 10.1038/s41698-023-00460-2 (PMC10567732; doi:10.1038/s41698-023-00460-2)
Supplement: Supplementary file 2 — REPORTING SUMMARY [file 41698_2023_460_MOESM2_ESM.pdf]

nature portfolio

Corresponding author(s): Ping-Pui Wong

Last updated by author(s): Jul 28, 2023

Reporting Summary

Nature Portfolio wishes to improve the reproducibility of the work that we publish. This form provides structure for consistency and transparency in reporting. For further information on Nature Portfolio policies, see our Editorial Policies and the Editorial Policy Checklist.

Statistics

For all statistical analyses, confirm that the following items are present in the figure legend, table legend, main text, or Methods section.

n/a	Confirmed	
☐	☒	The exact sample size (*n*) for each experimental group/condition, given as a discrete number and unit of measurement
☐	☒	A statement on whether measurements were taken from distinct samples or whether the same sample was measured repeatedly
☐	☒	The statistical test(s) used AND whether they are one- or two-sided<br>*Only common tests should be described solely by name; describe more complex techniques in the Methods section.*
☐	☒	A description of all covariates tested
☐	☒	A description of any assumptions or corrections, such as tests of normality and adjustment for multiple comparisons
☐	☒	A full description of the statistical parameters including central tendency (e.g. means) or other basic estimates (e.g. regression coefficient) AND variation (e.g. standard deviation) or associated estimates of uncertainty (e.g. confidence intervals)
☐	☒	For null hypothesis testing, the test statistic (e.g. *F*, *t*, *r*) with confidence intervals, effect sizes, degrees of freedom and *P* value noted<br>*Give P values as exact values whenever suitable.*
☒	☐	For Bayesian analysis, information on the choice of priors and Markov chain Monte Carlo settings
☒	☐	For hierarchical and complex designs, identification of the appropriate level for tests and full reporting of outcomes
☒	☐	Estimates of effect sizes (e.g. Cohen's *d*, Pearson's *r*), indicating how they were calculated

Our web collection on statistics for biologists contains articles on many of the points above.

Software and code

Policy information about availability of computer code

Data collection	All images were obtained by microscopy (Zeiss LSM 800 with airyscan, Nikon NI-U, Olympus IX71, MD ImageXpress Micro Confocal, Olympus FVMPE-RS).<br>FACS data were obtained by flow cytometry (Beckman CytoFLEX, Beckman MoFlo EQsB).<br>RT-PCR data were obtianed by Roche LightCycler 480 II.<br>Proteomics data were obtained by Orbitrap Exploris 480 System with lon Max Source.<br>Western Blot data were obtained by Mini Chemi 610.
Data analysis	Carl Zeiss ZEN 2011<br>ImageJ version 2.1.0<br>Graphpad Prism 8.0.1<br>cytoExpert 2.0<br>FlowJo v10<br>LightCycler 480 1.5.1<br>Kaplan-Meier plotter<br>Sage Capture (v1.2)

For manuscripts utilizing custom algorithms or software that are central to the research but not yet described in published literature, software must be made available to editors and reviewers. We strongly encourage code deposition in a community repository (e.g. GitHub). See the Nature Portfolio guidelines for submitting code & software for further information.

Data

Policy information about availability of data

All manuscripts must include a data availability statement. This statement should provide the following information, where applicable:

- Accession codes, unique identifiers, or web links for publicly available datasets
- A description of any restrictions on data availability
- For clinical datasets or third party data, please ensure that the statement adheres to our policy

The RNA-seq data generated in this study were deposited in the GEO under the accession number PRJNA905887. The proteomics data were deposited in the iProx database under the accession number PXD038343 (Web link: https://www.iprox.cn/page/PSV023.html;?url=1669253545524hzBg Password for reviewers: Jqru).

Research involving human participants, their data, or biological material

Policy information about studies with human participants or human data. See also policy information about sex, gender (identity/presentation), and sexual orientation and race, ethnicity and racism.

Reporting on sex and gender	No reporting on sex and gender were used in this study.
Reporting on race, ethnicity, or other socially relevant groupings	No reporting on race, ethnicity, or other socially relevant groupings were used in this study.
Population characteristics	Human head and neck squamous cell carcinoma (HNSCC) specimens were collected from patients who underwent surgery at the Sun Yat-sen Memorial Hospital with complete clinicopathological records. For the chemosensitivity study, we collected another cohort of the tumor biopsies and blood samples from HNSCC patients who received 3 cycles of cisplatin, 5'FU plus docetaxel based neoadjuvant chemotherapy (NACT). For drug response tracking experiments, the blood samples were collected from HNSCC patients before, during and after they received NACT treatment. Patients were obtained from Sun Yat-sen Memorial hospital (Guangzhou, China) with complete clinical data. The HNSCC patients who underwent curative freshly resected tissue enrolled for pericyte isolation were based on the following main criteria: (1) patients were absence of anticancer therapies prior to the operation. (2) No concurrent autoimmune disease, HIV, or syphilis.
Recruitment	Clinical samples were collected from Sun Yat-sen Memorial Hospital (Guangzhou, China) with complete clinical data. The biospecimens from each individual were collected at the time of surgery.
Ethics oversight	The collection of clinical specimens and related procedures were carried out with the approval of the internal review and ethnics committee of the Sun Yat-sen Memorial hospital (Ref no: SYSEC-KY-KS-2021-319).

Note that full information on the approval of the study protocol must also be provided in the manuscript.

Field-specific reporting

Please select the one below that is the best fit for your research. If you are not sure, read the appropriate sections before making your selection.

☒ Life sciences ☐ Behavioural & social sciences ☐ Ecological, evolutionary & environmental sciences

For a reference copy of the document with all sections, see nature.com/documents/nr-reporting-summary-flat.pdf

Life sciences study design

All studies must disclose on these points even when the disclosure is negative.

Sample size	The sample size of the Kmplotter database was determined by the number of tumor samples analysed with RNA sequencing. Sample size and number of independent experiments are stated in the figure legend. Three to more independent samples/experiments were used to perform statistical analysis. For our own patient cohorts, sample size was not statistically determined before collection. The determination of sample size is based on our experience and numerous publications, which is sufficient to generate statistically significant results.
Data exclusions	No data were excluded.
Replication	Each experiment was repeated at least 3 times independently, unless stated otherwise. The exact number of replicates were included in the legend or method section.
Randomization	Animal with similar ages and weight were randomly allocated to experimental groups before treated with corresponding drug combination or placebo. For experiments other than animals, the samples/cells were randomly allocated into different groups prior to treatment.
Blinding	For microscopy, flow cytometry, and other data collected by objective instruments, the researchers were not blinded to group allocation because they need to know which group each raw data corresponds to. But they were blinded during data analysis. The researchers who performed animal experiments was not blinded because they needed to know how to treat mice with different drug combination or placebo. But the researchers were blinded during the data analysis.

Reporting for specific materials, systems and methods

We require information from authors about some types of materials, experimental systems and methods used in many studies. Here, indicate whether each material, system or method listed is relevant to your study. If you are not sure if a list item applies to your research, read the appropriate section before selecting a response.

Materials & experimental systems

n/a	Involved in the study
☐	☒ Antibodies
☐	☒ Eukaryotic cell lines
☒	☐ Palaeontology and archaeology
☐	☒ Animals and other organisms
☒	☐ Clinical data
☒	☐ Dual use research of concern
☒	☐ Plants

Methods

n/a	Involved in the study
☒	☐ ChIP-seq
☐	☒ Flow cytometry
☐	☒ MRI-based neuroimaging

Antibodies

Antibodies used	anti-TGF (Cat no.#ab208156, Abcam (1 in 1000 dilution)), anti-EGFR (Cat no.#2232s, Cell Signalling Technology (1 in 1000 dilution)) , anti-p-EGFR (Cat no.#2236S , Cell Signalling Technology (1 in 1000 dilution)), anti-p-AKT (Cat no.#4060S, Cell Signalling Technology (1 in 1000 dilution)), anti-total AKT (Cat no.#4691S, Cell Signalling Technology (1 in 1000 dilution)), anti-total PI3K (Cat no.#20584-1-AP, Proteintech (1 in 1000 dilution)), anti-p-PI3K (Cat no.#AF3242-50, Affinity (1 in 1000 dilution)), anti-p-Src (Cat no.#2101S, Cell Signalling Technology (1 in 1000 dilution), anit-total Src (Cat no.#2109S, Cell Signalling Technology (1 in 1000 dilution), anti-cleaved caspase-3 (Cat no.#9661S, Cell Signalling Technology (1 in 1000 dilution)), anti-p-p53(Cat no.#9284S, Cell Signalling Technology (1 in 1000 dilution)), total p53(Cat no.#sc-126, Santa Cruz (1 in 1000 dilution), p-p65(Cat no.#3033S, Cell Signalling Technology (1 in 1000 dilution)), total p65(Cat no.#8242S, Cell Signalling Technology (1 in 1000 dilution)). β-actin (Cat no.#sc-47778, Santa Cruz (1 in 5000 dilution)
Validation	All antibodies were validated by the mamufacturer.

Eukaryotic cell lines

Policy information about cell lines and Sex and Gender in Research

Cell line source(s)	FaDu and Tu686 cells
Authentication	Cell lines were used as provided commercially and no additional identification was performed.
Mycoplasma contamination	No mycoplasma contamination was detected during the program.
Commonly misidentified lines (See ICLAC register)	No commonly misidentified lines were used in the project.

Animals and other research organisms

Policy information about studies involving animals; ARRIVE guidelines recommended for reporting animal research, and Sex and Gender in Research

Laboratory animals	4-6 weeks old female C57/BLk6 and nude mice purchased from the Guangdong Medical Science Experiment Center were used in this study. Mice were allowed to acclimate to local conditions for 1 week and housed at 22 ±2 celcius degree, humidity 50 ±10% under a 12h dark/12h light cycle with adequate food and water.
Wild animals	No wild animals were used in this study.
Reporting on sex	No reporting on sex were used in this study.
Field-collected samples	No field-collected samples were used in this study.
Ethics oversight	All animal procedures were approved by the Institutional Animal Care and Use Committee (IACUC) of Sun Yat-sen University and adhered to the ARRIVE guidelines.

Note that full information on the approval of the study protocol must also be provided in the manuscript.

Flow Cytometry

Plots

Confirm that:

☒ The axis labels state the marker and fluorochrome used (e.g. CD4-FITC).

☒ The axis scales are clearly visible. Include numbers along axes only for bottom left plot of group (a 'group' is an analysis of identical markers).

☒ All plots are contour plots with outliers or pseudocolor plots.

☒ A numerical value for number of cells or percentage (with statistics) is provided.

Methodology

Sample preparation	Sample preparation steps are described in the materials and methods setion.
Instrument	Beckman CytoFLEX, Beckman MoFlo EQsB
Software	CytoExpert 2.0, FlowJo V10
Cell population abundance	The percentage of viable cells, early apoptotic cells, and late apoptotic cells was determined based on the lower quadrant (Q4), lower right quadrant (Q3), and upper quadrant (Q2), respectively.
Gating strategy	The red number indicates the percentage of apoptotic cells in each group.

☒ Tick this box to confirm that a figure exemplifying the gating strategy is provided in the Supplementary Information.

Magnetic resonance imaging

Experimental design

Design type	HNSCC patients response to chemotherapeutic agents was monitored by performing magnetic resonance imaging (MRI) before and after the NACT treatment
Design specifications	chemoresistant HNSCC patients were those with progressive disease (PD) or stable disease (SD), while the patients with complete response or partial response were classified as chemosensitive patients
Behavioral performance measures	sensitive patients (CR: complete response, PR: partial response) or resistant patients (SD: stable disease and PD: progressive disease)), was monitored and evaluated effectively by magnetic resonance imaging (MRI) before and after the NACT treatment

Acquisition

Imaging type(s)	Magnetic resonance imaging (MRI) of a chemo-sensitive or chemo-resistant patient before and after receiving neoadjuvant chemotherapy (NACT) treatment (3 cycles of treatment). Red line indicates the position of tumor. TNM staging: T stands for tumor, N for lymph node metastasis and M for metastasis. Y stands for year. T1WI refers to T1 weighted images and T1WI+C stands for contrast enhanced T1WI.
Field strength	n/a
Sequence & imaging parameters	n/a
Area of acquisition	n/a

Diffusion MRI ☐ Used ☒ Not used

Preprocessing

Preprocessing software	n/a
Normalization	n/a
Normalization template	n/a
Noise and artifact removal	n/a
Volume censoring	n/a

Statistical modeling & inference

Model type and settings	n/a
Effect(s) tested	n/a

Specify type of analysis: ☐ Whole brain ☐ ROI-based ☐ Both

Statistic type for inference (See Eklund et al. 2016)	n/a
Correction	n/a

Models & analysis

n/a	Involved in the study
☒	☐ Functional and/or effective connectivity
☒	☐ Graph analysis
☒	☐ Multivariate modeling or predictive analysis
